# Linking Host Provenance, Microbial Assembly, and Environmental Legacy Effects in the Root-Associated Microbiome of *Tripterygium wilfordii* Hook f.

**DOI:** 10.3390/microorganisms14081694

**Published:** 2026-08-01

**Authors:** Dongdong Ma, Shuzhen He, Shaoying Li, Li Lin, Yihui Wang, Lei Feng, Zhaoshou Lin, Chengzhen Wu, Ping Song

**Affiliations:** 1 College of Forestry, Fujian Agriculture and Forestry University, Fuzhou 350002, China; 12304028009@fafu.edu.cn (D.M.); 52304022025@fafu.edu.cn (S.H.); 12304054007@fafu.edu.cn (S.L.); 12427054004@fafu.edu.cn (L.L.);; 2 College of Resources and Environment, Fujian Agriculture and Forestry University, Fuzhou 350002, China; 3 Datian Taoyuan State Forest Farm of Fujian Province, Sanming 366031, China; tylcps513@163.com; 4 Fujian Provincial Key Laboratory of Forest Ecosystem Processing and Management, Fujian Agriculture and Forestry University, Fuzhou 350002, China; fjwcz@126.com

**Keywords:** *Tripterygium wilfordii*, geographic provenance, root-associated microbiome, community diversity and structure, climatic signatures

## Abstract

Plant-associated microbiomes play critical roles in regulating plant growth, nutrient acquisition, and stress adaptation. However, whether geographically distinct plant provenances retain heritable signatures that influence microbiome assembly under uniform environmental conditions remains poorly understood. In this study, 16S rRNA amplicon sequencing was employed to characterize root-associated bacterial communities of clonally propagated *Tripterygium wilfordii* derived from nine provenances and cultivated under identical soil conditions, with additional sampling from native populations. Host provenance significantly affected community composition, explaining 27.0% and 32.6% of the variation in rhizosphere and endophytic communities, respectively. Rhizosphere bacterial diversity differed significantly among provenances, whereas endophytic communities showed stronger differentiation in community structure and deterministic host selection. Although native plants exhibited lower bacterial diversity, they recruited more potential plant growth-promoting bacteria compared with transplanted plants. Environmental factors, particularly historical mean annual precipitation and longitude of native habitats, significantly influenced community diversity and structure. Distance–decay patterns demonstrated that root-associated bacterial communities retained climatic imprints from their native provenances despite cultivation under common-garden conditions. This study provides evidence that plant provenance can leave persistent microbial signatures and highlights the importance of integrating host genetic background and microbiome characteristics to understand ecological adaptation and improve germplasm utilization in medicinal plants.

## 1. Introduction

Plant-associated microorganisms are increasingly recognized as essential components that shape plant adaptation, growth, and ecological performance [1]. Among these microbial partners, rhizosphere and endophytic communities play pivotal roles in nutrient acquisition, phytohormone regulation, stress tolerance, and disease resistance [2,3]. These microorganisms can enhance plant nutrient acquisition through processes such as biological nitrogen fixation, phosphorus solubilization, and micronutrient mobilization [4,5]. For example, members of the genera *Rhizobium* and *Bacillus* increase nitrogen availability through atmospheric nitrogen fixation, whereas phosphate-solubilizing bacteria improve phosphorus accessibility by releasing organic acids [6,7,8,9]. In addition, plant-associated microorganisms regulate host physiology by producing phytohormones and modulating gene expression. Under environmental stress, beneficial microorganisms can activate signaling pathways involving abscisic acid, salicylic acid, and ethylene, thereby enhancing plant resilience to adverse environments [10]. Microbial synthesis of indole-3-acetic acid (IAA), gibberellins, and other growth-promoting compounds further stimulates root development and nutrient uptake [11,12]. Moreover, microorganisms contribute to plant health through direct pathogen antagonism and by inducing systemic resistance [13]. In medicinal plants, associated microorganisms can also regulate secondary metabolic pathways and influence the accumulation of bioactive compounds such as terpenoids and flavonoids [14,15,16,17,18]. Therefore, plant-associated microbiomes are regarded as critical determinants of plant phenotypes and ecological fitness.

The assembly of plant-associated microbial communities is regulated by a complex interplay of environmental factors, soil properties, and host characteristics. Among these factors, host genetic variation has emerged as a key driver of microbiome assembly. Increasing evidence indicates that different plant genotypes selectively recruit distinct microbial taxa from shared environmental microbial reservoirs, resulting in genotype-specific microbial community structures [19]. In barley, significant differences in rhizosphere bacterial and fungal communities have been identified among host genotypes, with elite cultivars recruiting a greater abundance of beneficial microorganisms than ancestral genotypes [20]. These genotype-associated microbial communities are positively correlated with root biomass and can contribute to the suppression of root lesion nematodes through the activation of salicylic acid-mediated defense pathways [20]. Similarly, genotype-dependent microbial recruitment has been documented in pea, tomato, and *Arabidopsis thaliana*, where host genetic variation influences microbial community composition, the abundance of key microbial taxa, and ultimately plant performance [21,22,23]. Notably, genotype effects can persist across generations, as microbial taxa associated with specific host genotypes can remain detectable following repeated microbial community transfers [22]. Host-mediated microbial selection is driven primarily by root exudation and immune regulation. Plant genotypes exhibit substantial variation in the quantity and composition of root exudates, thereby generating distinct ecological niches that influence microbial recruitment and colonization. For instance, nitrogen-efficient rapeseed genotypes selectively enrich members of the family Bacillaceae through the secretion of kaempferol, thereby enhancing nitrogen acquisition and rhizosphere biofilm formation [24]. Similarly, compared with the landrace cultivar Chevallier, the modern barley cultivar NFC Tipple recruits distinct *Pseudomonas* populations by increasing the secretion of hexose compounds [25]. In addition, plant immune systems regulate microbiome assembly through the recognition of microbe-associated molecular patterns and microbial effectors [26,27]. Collectively, these findings indicate that host genetic variation not only determines microbial community composition but also establishes feedback mechanisms that influence plant health, productivity, and adaptation.

*Tripterygium wilfordii* Hook f., a woody vine of medicinal importance widely distributed across southern China, is renowned for its anti-inflammatory, immunomodulatory, and antitumor activities [28,29,30]. More than 500 natural compounds, including sesquiterpenes, diterpenes, alkaloids, and polysaccharides, have been isolated from this species, many of which exhibit important pharmacological properties and have been widely used in clinical practice [31]. Previous studies have demonstrated considerable variation among geographically distinct *T. wilfordii* populations in growth performance, physiological traits, and the accumulation of medicinal compounds [32,33]. Establishing a germplasm repository by collecting plant clones from different geographic origins is an important approach for screening superior genotypes with desirable agronomic and medicinal traits. However, conventional germplasm evaluation and breeding programs have primarily emphasized plant phenotypic traits, while the role of associated microorganisms has been overlooked. As plant phenotypic traits are shaped not only by host genetic variation but also substantially influenced by the associated microbiome, it is essential to investigate plants from different provenances from a holobiont perspective that integrates both host characteristics and their microbial partners.

Previous study has characterized culturable root-associated bacterial communities of *T. wilfordii* from three geographic provenances and revealed provenance-associated differences in bacterial taxonomic composition [34]. However, culture-dependent approaches capture only a fraction of microbial diversity, and the broader patterns underlying root-associated microbiome assembly, including uncultured microbial taxa and the ecological factors driving community differentiation among provenances, remain largely unexplored. In this study, we hypothesized that genetic variation among *T. wilfordii* provenances drives the differentiation of root-associated bacterial communities and that such differentiation is associated with the geographic distance among their host origins. To test this hypothesis, we used 16S rRNA amplicon sequencing to characterize the diversity, composition, and co-occurrence network structures of root-associated bacterial communities in clonally propagated plants originating from different provenances and cultivated under identical soil conditions. We further quantified the relative contributions of host provenance, soil physicochemical properties, and environmental factors to microbial community variation and identified potential provenance-specific bacterial taxa. This study aims to determine whether root-associated bacterial communities reflect provenance-related genetic differentiation under clonal propagation and to elucidate the potential drivers shaping these patterns. That could provide the basis for understanding the adaptive mechanisms of *T. wilfordii* and informing strategies for germplasm conservation and utilization.

## 2. Materials and Methods

### 2.1. Sample Collection

Nine provenances of *T. wilfordii* (Supplementary Table S1) were sampled from the Taoyuan State Forest Farm, Datian County, Fujian Province, China. Four individual plants from each provenance were collected as biological replicates. These plants were originally established in a provenance conservation plantation in 2013 through cutting propagation from nine geographic regions, including Fujian (117°56′28″ E, 25°48′22″ N), Anhui (117°39′41″ E, 29°50′34″ N), Zhejiang (119°40′06″ E, 28°19′46″ N), Yunnan (100°13′49″ E, 24°39′43″ N), Sichuan (101°58′38″ E, 27°01′56″ N), Guangxi (108°23′51″ E, 24°35′29″ N), Hunan (112°31′35″ E, 27°40′59″ N), Hubei (113°57′28″ E, 29°11′29″ N), and Guizhou (108°05′26″ E, 26°19′19″ N), China. The Taoyuan State Forest Farm, located in the western Daiyun Mountain region of central Fujian (117°34′ E, 25°51′ N), experiences a subtropical monsoon climate characterized by moderate temperatures, high solar radiation, abundant precipitation, and predominantly red and yellow soils. To compare microbial communities between introduced provenances and natural populations, additional root samples were collected from indigenous *T. wilfordii* populations in Hubei Province (113°57′ E, 29°11′ N) and Hunan Province (112°31′ E, 27°40′ N), with eight biological replicates obtained from each location. The sampling regions in Hubei and Hunan are characterized by subtropical monsoon climates with red and yellow-brown soils in Hubei and paddy soils in Hunan, respectively. Detailed information on altitude, climate, and soil physicochemical properties of the three sampling sites has been reported previously by Song et al. [34]. Root systems with adhering rhizosphere soil were collected from the 0–20 cm soil layer. All samples were immediately placed on ice and transported to the laboratory for further processing.

### 2.2. Preparation of Root Samples

Root segments with adhering rhizosphere soil were placed into sterile 100 mL centrifuge tubes containing sterile distilled water and vortexed for 1 min. This procedure was repeated several times until the visible soil was removed from the root surface. The soil suspension was centrifuged at 6000 rpm and 4 °C for 8 min using a refrigerated high-speed centrifuge (HC-2518R, Anhui Zhongke Zhongjia Scientific Instrument Co., Ltd., Hefei, China), and the collected pellets were used as the rhizosphere soil sample for DNA extraction. Remaining root tissues were surface-sterilized with 2% sodium hypochlorite for 20 s, followed by 70% ethanol for 10 min, and rinsed five times with sterile distilled water. Sterilized root tissues were flash-frozen in liquid nitrogen and stored at −80 °C for endophytic microbial analysis.

### 2.3. 16S rRNA Gene Amplicon Sequencing

Genomic DNA was extracted using the FastDNA Spin Kit for Soil (MP Biomedicals, Irvine, CA, USA) following the manufacturer’s instructions. DNA quality was assessed via 1.0% agarose gel electrophoresis. The V3–V4 region of the bacterial 16S rRNA gene was amplified using barcoded primers 338F and 806R in a 20 μL reaction system with the TransStart FastPfu DNA Polymerase kit (TransGen Biotech, Beijing, China). The reaction mixture contains 4 μL of 5× FastPfu Buffer, 2 μL of 2.5 mM dNTPs, 0.8 μL of each of forward and reverse primer (5 μM), 0.4 μL of FastPfu Polymerase, 0.2 μL of BSA, and 10 ng of template DNA, with ddH_2_O added to a final volume of 20 μL. Amplified products were verified by gel electrophoresis, purified, and used for library construction with the Illumina TruSeq DNA Sample Preparation Kit (Illumina, San Diego, CA, USA). Libraries were quantified using the QuantiFluor™-ST system (Promega, Madison, WI, USA) and sequenced on an Illumina MiSeq platform (Shanghai Majorbio Bio-Pharm Technology Co., Ltd., Shanghai, China).

Paired-end reads were assembled based on overlap using FLASH (v1.2.11), and quality filtering was performed using Trimmomatic (v0.36). Operational taxonomic units (OTUs) were clustered at 97% sequence similarity using USEARCH (v9.2). Taxonomic classification was performed against the SILVA database.

### 2.4. Soil Physicochemical Properties

Soil samples were collected from vicinity of roots, and visible roots and plant residues were removed. The samples were air-dried at room temperature, ground, and sieved through 1 mm and 0.149 mm meshes prior to the physicochemical analysis [35]. All measurements were performed with three technical replicates for each sample, and reagent blanks were included during chemical determinations. Soil pH was measured using a glass electrode (FE22, Mettler-Toledo Instruments (Shanghai) Co., Ltd., Shanghai, China) in a 1:2.5 (*w*/*v*) soil-to-water suspension after shaking for 30 min [36]. Total carbon (TC) and total nitrogen (TN) were measured using an elemental analyzer (EA3000, EuroVector S.p.A., Pavia, Italy) with finely ground soil samples (<0.149 mm) [37]. Total phosphorus (TP) was determined following H_2_SO_4_–HClO_4_ digestion and molybdenum–antimony colorimetric determination by UV spectrophotometry (TU-1810, Beijing Purkinje General Instrument Co., Ltd., Beijing, China) [38]. Available phosphorus (AP) was extracted with 0.5 mol L^−1^ NaHCO_3_ solution (soil:solution ratio = 1:25, *w*/*v*) and determined using the molybdenum–antimony colorimetric method [38]. Available potassium (AK) was extracted with 1 mol L^−1^ NH_4_OAc solution and measured by flame photometry (PinAAcle 900Z, PerkinElmer Instruments (Suzhou) Co., Ltd., Suzhou, China). Soil organic matter (OM) was determined using the potassium dichromate–sulfuric acid oxidation method. Available nitrogen (AN) was determined using the alkaline diffusion method after incubation with NaOH at 40 °C for 24 h. The determination procedures for AK, OM, and AN followed established methods described by Wang et al. [39]. The detailed values of soil physicochemical properties for each provenance are provided in Supplementary Table S2.

### 2.5. Statistical Analysis

All statistical analyses were conducted in R (version 4.3.2) using relevant packages. Hierarchical clustering analysis was performed with the pvclust package (v2.2.0) based on Bray–Curtis dissimilarity and Ward’s linkage method [40]. Approximately unbiased (AU) *p*-values were calculated using multiscale bootstrap resampling, with AU > 95% indicating robust clustering. Bacterial community source tracking was conducted using the fast expectation-maximization for microbial source tracking (FEAST) algorithm [41], which estimates the contributions of potential sources to sink communities through an expectation-maximization (EM) framework with 1000 iterations under default parameters. Procrustes analysis was performed to evaluate the similarity between rhizosphere and endophytic bacterial community structures using non-metric multidimensional scaling (NMDS) ordination based on Bray–Curtis dissimilarity. Differentially abundant taxa were identified using linear discriminant analysis effect size (LEfSe) analysis implemented in the ggtree package (v3.16.3) with Kruskal–Wallis tests. Alpha diversity indices were calculated using the vegan package (v2.7.1). Principal coordinate analysis (PCoA) was performed to visualize variation in bacterial taxonomic composition and functional profiles. Taxonomic and functional dissimilarity were calculated using weighted UniFrac/Bray–Curtis and Bray–Curtis distances, respectively, followed by permutational multivariate analysis of variance (PERMANOVA) using the adonis function in the vegan package. The neutral community model (NCM) of Sloan et al. [42] was employed to quantify the relative contribution of neutral processes to community assembly, with computations performed using the Hmisc (v5.2.3), minpack.lm (v1.2.4), and getopt packages (v1.20.3). Ecological assembly processes were inferred using the iCAMP package (v1.5.12) based on βNTI and RCbray metrics [43,44]. Prior to network construction, rare OTUs with relative abundance < 0.01% and occurrence frequencies in <20% of samples were removed. Spearman correlations between OTUs were calculated using the WGCNA package (v1.73) (corAndPvalue function). Co-occurrence networks were constructed using different thresholds: |r| > 0.60 with adjusted *p* < 0.05 for general datasets, and |r| > 0.70 with adjusted *p* < 0.01 for provenance-specific datasets. Networks were visualized in Gephi 0.10.1 (https://gephi.org/, accessed on 7 May 2026) using the Fruchterman–Reingold layout. Topological properties including modularity, average degree, density, average clustering coefficient, average path length, network density and network diameter, were calculated in Gephi. Network robustness was evaluated using the ggClusterNet package (v2.0) by simulating random node removal and assessing changes in network connectivity. Keystone taxa were identified based on within-module connectivity (Zi) and among-module connectivity (Pi), and were categorized as module hubs, network hubs, connectors, or peripherals according to the criteria Zi > 2.5 and Pi > 0.62. Additional keystone taxa were identified using the SPEC-OCCU framework with specificity and occupancy thresholds of ≥0.7 [45,46,47]. Environmental drivers of microbial diversity were analyzed using Spearman correlations, Mantel tests, and climatic variables obtained from WorldClim v2.1 (https://worldclim.org/, accessed on 6 May 2026). Elevation data (m a.s.l.) were extracted based on geographic coordinates using the elevatr package (v0.99.1), with elevation values derived from digital elevation model (DEM) data provided through Amazon Web Services (AWS). Random forest analysis was conducted using the randomForest (v4.7.1.2) and rfPermute packages (v2.5.5). Distance–decay relationships (DDRs) were evaluated using ordinary least squares regression between geographic/climatic distances and Bray–Curtis dissimilarity. Redundancy analysis (RDA) and variation partitioning analysis (VPA) were performed using the vegan package, and the individual contributions of explanatory variables were quantified using the rdacca.hp package (v1.1.1). Model selection was conducted using MuMIn (dredge function) based on corrected Akaike information criterion (AICc) (ΔAICc < 2). Structural equation modeling (SEM) was performed using the plspm package (v0.6.0), based on a partial least squares path modeling (PLS-PM) approach. This variance-based SEM approach was used to evaluate the direct and indirect effects of provenance geographic location, climatic variables, and soil physicochemical properties on microbial diversity, community composition, and network complexity [48]. Functional prediction of microbial communities was performed using FAPROTAX. Differences in predicted microbial functions among communities were evaluated using PCoA and PERMANOVA based on Bray–Curtis distances in the vegan package.

## 3. Results

### 3.1. Biogeographic Patterns of Microbial Communities

**Figure 1:**
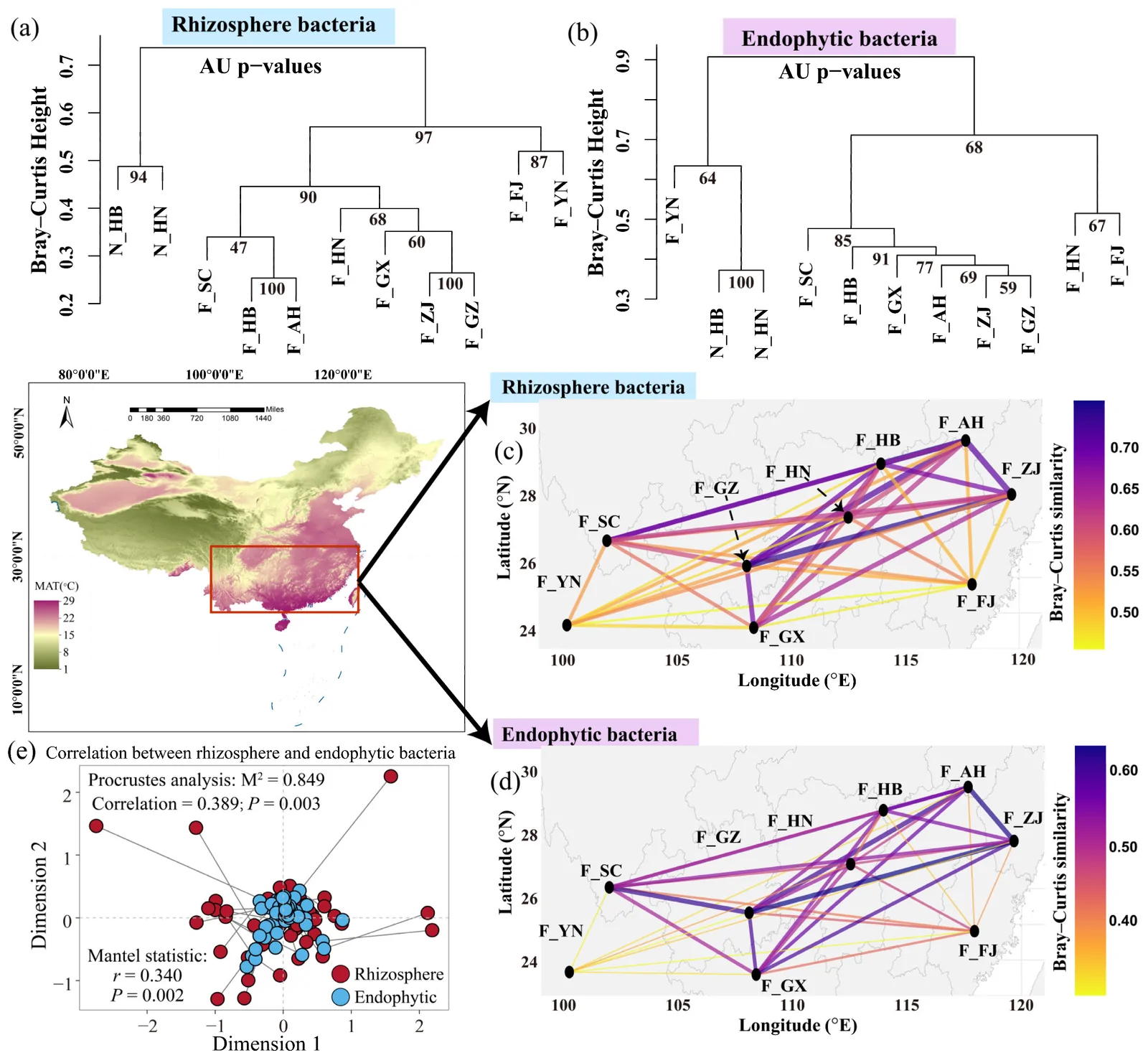
(**a**,**b**) Community similarity and relationships among root-associated bacterial communities of *T. wilfordii* provenances. (**a**,**b**) Ward hierarchical clustering of samples based on OTU composition using Bray–Curtis dissimilarity. Approximately unbiased (AU) *p*-values are indicated on the dendrogram branches, with AU > 95% considered strong support for clustering; (**c**,**d**) compositional similarity network among samples constructed using Bray–Curtis similarity, where link thickness (or color intensity) represents the degree of community similarity between samples; (**e**) Procrustes analysis comparing rhizosphere and endophytic bacterial communities based on non-metric multidimensional scaling (monoMDS) ordinations derived from Bray–Curtis dissimilarity matrices. Rhizosphere and endophytic communities are indicated by different colors as shown in the legend, and connecting lines link corresponding samples between the two compartments.

Hierarchical clustering based on Bray–Curtis dissimilarity revealed that cultivation environment strongly influenced on root-associated microbial communities, with samples from the same cultivation site tending to cluster together, particularly in rhizosphere bacterial communities (Figure 1). Further analysis showed that both rhizosphere and endophytic bacterial communities of *T. wilfordii* from different provenances were classified into four major clusters (Figure 1a,b). In the Fujian common garden, Bray–Curtis similarities among rhizosphere bacterial communities ranged from 0.48 to 0.75 (Figure 1c,d), indicating substantial variation in community composition among provenances. The highest similarity was observed between F_HB and F_AH (0.75). F_ZJ showed high similarity with most other provenances, whereas F_YN and F_FJ exhibited relatively low similarity to other groups (Figure 1c). A similar pattern was observed in endophytic bacterial communities (Figure 1d), suggesting that host provenance significantly influences both rhizosphere and endophytic microbial assembly under identical environmental conditions. Procrustes analysis demonstrated a significant concordance between rhizosphere and root endophytic microbial communities (M^2^ = 0.849, r = 0.389, *p* = 0.003). Mantel tests further confirmed a significant positive correlation between community dissimilarity matrices of rhizosphere and endophytic bacterial communities (Figure 1e).

### 3.2. Microbial Source Tracking Analysis

**Figure 2:**
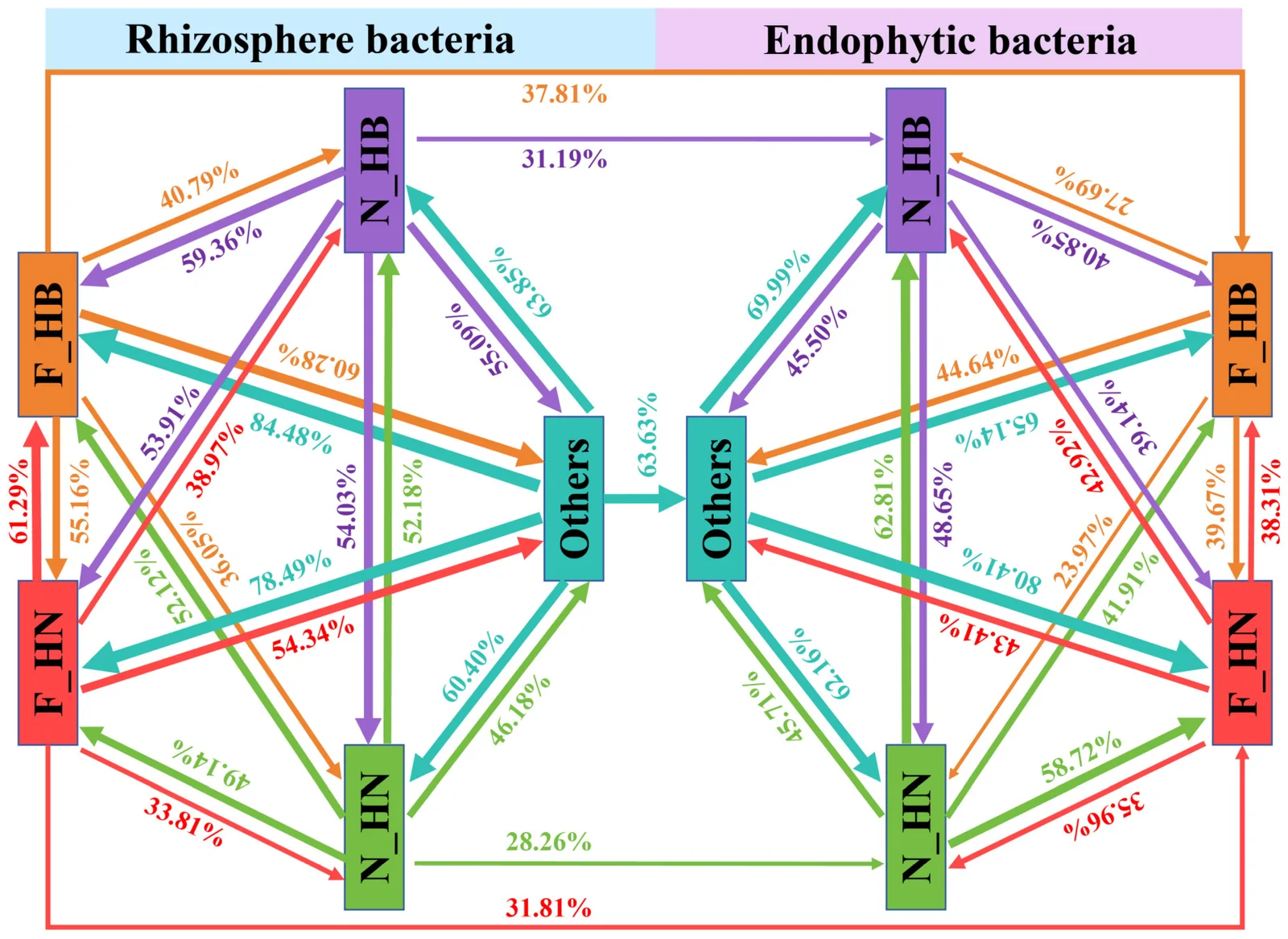
Source–sink relationships inferred by FEAST. Arrows indicate the direction of source-to-sink relationships, and percentages represent the relative contribution of each source to the sink communities.

FEAST analysis indicated the distinctive source contributions of provenance-associated microbial communities (Figure 2). In the rhizosphere, approximately 78.49% of the microbial community in F_HN originated from other provenances cultivated at the same site, of which 55.16% was attributed to F_HB. In contrast, contributions from native microbial sources were relatively limited to F_HN (49.14%). Similarly, 84.48% of the F_HB rhizosphere community was derived from other co-cultivated provenances, including 61.29% from F_HN, whereas contributions from native F_HB were relatively lower (59.36%). In endophytic communities, 80.41% of the F_HN microbiome originated from other cultivated provenances, with 58.72% attributed to native N_HN, while rhizosphere-derived sources contributed the least (31.81%). For F_HB, 65.14% of the endophytic community originated from other provenances, including 41.91% from N_HN and 40.85% from N_HB, whereas rhizosphere contributions remained relatively low (37.81%). Notably, native populations N_HN and N_HB also showed limited contribution to rhizosphere communities (28.26% and 31.19%, respectively), suggesting restricted microbial dispersal from native populations to non-native provenances.

### 3.3. Community Diversity and Assembly Processes

**Figure 3:**
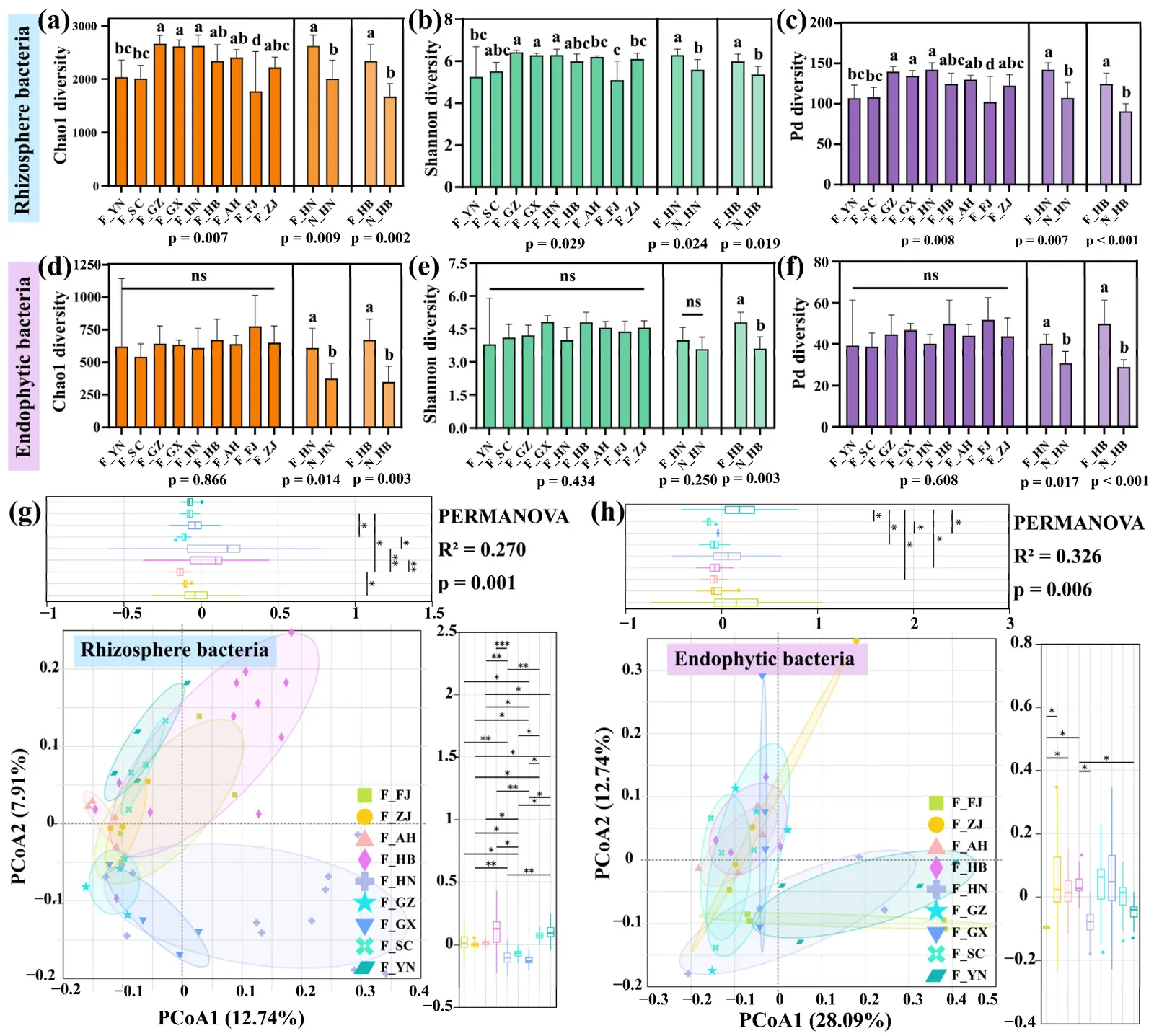
Diversity patterns of bacterial communities: (**a**–**f**) Alpha diversity indices of root-associated bacterial communities. Statistical significance was assessed using one-way ANOVA with Bonferroni correction or Student’s *t*-test for pairwise comparisons. Different lowercase letters indicate significant differences among groups (*p* < 0.05) and ns represents no significant difference (*p* > 0.05). (**g**,**h**) Principal coordinates analysis (PCoA) based on weighted UniFrac distances. Asterisks represent significant differences (* *p* < 0.05, ** *p* < 0.01, and *** *p* < 0.001).

To evaluate the diversity of rhizosphere and endophytic microbiomes associated with *T. wilfordii*, α-diversity indices, including the Chao1, Shannon, and phylogenetic diversity (PD) indices, were calculated at the OTU level for nine provenances from three geographic regions (Figure 3). Significant differences in rhizosphere bacterial α-diversity were observed among nine provenances cultivated in the common garden. For all three α-diversity indices (Chao1: 1774.82–2666.64; Shannon: 5.09–6.43; PD: 102.45–142.28), F_HN (Chao1: 2625.03; Shannon: 6.30; PD: 142.28), F_GX (Chao1: 2616.25; Shannon: 6.29; PD: 134.73), and F_GZ (Chao1: 2666.64; Shannon: 6.43; PD: 139.92) consistently belonged to the group with the highest average values. These results indicated that provenance variation strongly influenced rhizosphere bacterial richness and diversity. The populations N_HB and N_HN from their native habitats exhibited significantly lower Chao1 (N_HB: 1673.74; N_HN: 2006.76), Shannon (N_HB: 5.37; N_HN: 5.59), and PD (N_HB: 90.76; N_HN: 107.24) values than their corresponding provenances F_HB (Chao1: 2341.46; Shannon: 6.00; PD: 124.77) and F_HN cultivated outside their native habitats, suggesting reduced richness and diversity in indigenous rhizosphere bacterial communities (Figure 3a–c). For endophytic bacterial communities, no significant differences in Chao1, Shannon, or PD indices were detected among the nine provenances, indicating that provenance had relatively limited effects on endophytic bacterial richness and diversity. Nevertheless, N_HN (Chao1: 376.31; Shannon: 3.59; PD: 30.88) and N_HB (Chao1: 349.52; Shannon: 3.60; PD: 29.04) consistently showed lower α-diversity values than F_HN (Chao1: 610.93; Shannon: 4.00; PD: 40.28) and F_HB (Chao1: 673.18; Shannon: 4.81; PD: 49.87), respectively (Figure 3d–f).

PCoA based on weighted UniFrac distances revealed significant differences in both rhizosphere (*R*^2^ = 0.270, *p* = 0.001) and endophytic (*R*^2^ = 0.326, *p* = 0.006) bacterial community structures among the nine provenances cultivated in the common environment (Figure 3g,h). For rhizosphere communities, the first two principal coordinates explained 12.74% and 7.91% of the total variation, respectively (Figure 3g). Except for F_YN and F_ZJ, most provenances were significantly separated along the first principal coordinate axis (PCoA1, *p* < 0.05). In addition, all provenances showed significant separation along the second principal coordinate axis (PCoA2, *p* < 0.05). Similarly, for endophytic communities, PCoA1 and PCoA2 explained 28.09% and 12.74% of the variation, respectively (Figure 3h), and most provenances exhibited significant separation along one or both axes. The explanatory power of provenance was greater for endophytic bacteria, indicating a stronger host-genotype effect on endophytic than rhizosphere bacterial communities. In addition, both microhabitat *R*^2^ = 0.135, *p* = 0.001) and cultivation site (rhizosphere: *R*^2^ = 0.220, *p* = 0.001; endosphere: *R*^2^ = 0.116, *p* = 0.001) significantly affected bacterial community composition (Supplementary Figure S1a–c).

Both the rhizosphere and endophytic bacterial communities of nine provenances at the identical growing environment fit the NCM well. The model explained a substantial proportion of variation in both rhizosphere (*R*^2^ = 0.8088) and endophytic bacterial communities (*R*^2^ = 0.7229) (Supplementary Figure S1d,e), suggesting that stochastic processes played a major role in bacterial community assembly. However, the higher migration rate (m) of rhizosphere bacteria (0.4075) than endophytic bacteria (0.2298) indicated stronger dispersal limitation within plant tissues. The relative contributions of ecological processes inferred by iCAMP further supported the predominance of stochastic assembly mechanisms (Supplementary Figure S1f,g). Drift and other stochastic processes (DR) represented the largest proportion across all provenances in both rhizosphere and endophytic bacterial communities. In rhizosphere communities, DR accounted for approximately 36–65% of community assembly, followed by homogeneous selection (HoS) and dispersal limitation (DL). Endophytic communities displayed a similar pattern, although the contribution of homogeneous selection was generally higher than that observed in rhizosphere communities. In contrast, heterogeneous selection (HeS) and homogeneous dispersal (HD) contributed relatively little to community assembly in either habitat. Overall, iCAMP analysis revealed that bacterial community assembly in root-associated niches was primarily governed by stochastic processes, while deterministic host filtering appeared to play a greater role in shaping endophytic communities.

### 3.4. Taxonomic Composition of Microbial Communities

**Figure 4:**
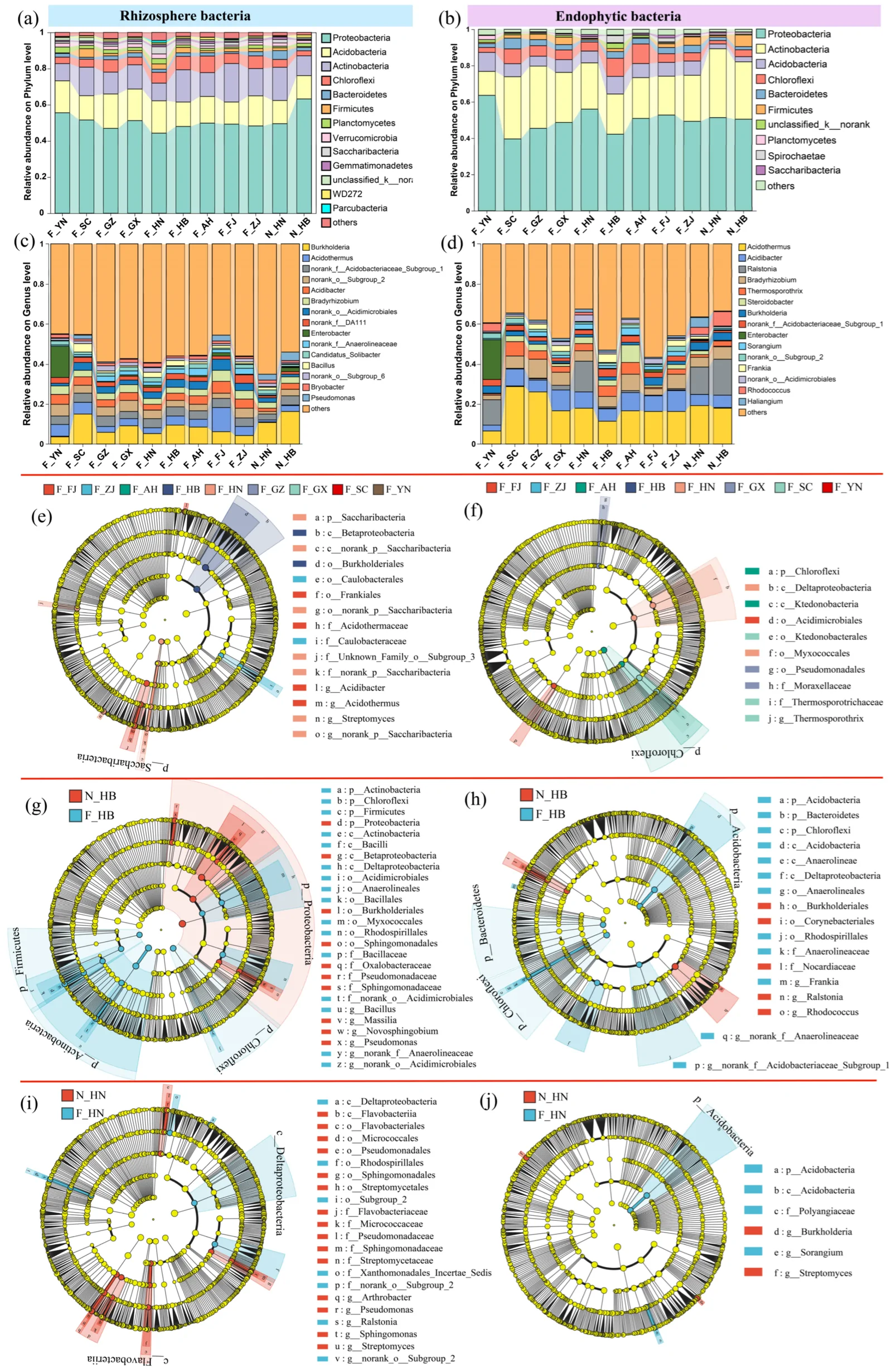
Bacterial community composition and differential analysis across different plant provenances: (**a**,**b**) Relative abundance of microbial communities with a relative abundance > 1% at the phylum level. (**c**,**d**) Relative abundance of the top 15 microbial taxa at the genus level. (**e**–**j**) Differentially enriched bacterial taxa identified among provenances. The cladograms display taxonomic hierarchies from phylum (inner circles) to genus (outer circles). Different colors represent different plant provenances. Taxa highlighted in the cladogram indicate significantly enriched groups in each provenance, as determined by the Kruskal–Wallis test (*p* < 0.05) with a logarithmic LDA score > 4.

The taxonomic composition of root-associated bacterial communities differed among provenances (Figure 4). In rhizosphere, Proteobacteria was consistently the dominant phylum across all samples, accounting for 44.36–63.21% of total sequences. Other abundant phyla included Acidobacteria (12.22–19.03%), Actinobacteria (9.52–21.30%), and Chloroflexi (2.78–9.16%) (Figure 4a). At the genus level, rhizosphere communities were mainly composed of *Burkholderia* (3.70–16.35%), *Acidothermus* (1.13–11.97%), Acidobacteriaceae_Subgroup_1 (2.84–5.25%), norank_o__Subgroup_2 (1.63–5.84%), and *Acidibacter* (1.53–4.77%). Notably, *Enterobacter* was highly enriched in the F_YN provenance, reaching a relative abundance of 15.68%, substantially higher than in other samples (Figure 4c). SPEC–OCCU analysis identified 128 provenance-specialized OTUs in rhizosphere communities (Supplementary Figure S2a and Table S3). F_GX (37 OTUs) and F_HN (31 OTUs) contained the relative larger number of specialized taxa. Out of the specialized 128 OTUs, 32 belonged to Proteobacteria (25%), 25 belonged to Chloroflexi (19.5%), and 12 belonged to Planctomycetes (9.4%). LEfSe analysis identified 15 bacterial biomarkers among rhizosphere communities of nine different provenances (Figure 4e). Among the provenances, F_HN exhibited the highest number of bacterial biomarkers (7), followed by F_FJ (4). The major biomarkers included Frankiales (F_FJ), *Acidothermus* (F_FJ), *Acidibacter* (F_FJ), Caulobacteraceae (F_ZJ), Burkholderiales (F_HB), *Streptomyces* (F_HN), *Saccharibacteria* (F_HN). Comparisons of the rhizosphere bacterial communities between native and non-native plants (N_HB vs. F_HB, N_HN vs. F_HN) revealed that the principal bacterial biomarkers associated with native plants were Burkholderiales, *Pseudomonas*, *Massilia*, *Novosphingobium*, *Sphingomonas*, *Arthrobacter*, and *Streptomyces* (Figure 4g,i). In contrast, the predominant biomarkers associated with non-native plants included Actinobacteria, Chloroflexi, Acidimicrobiales, *Bacillus*, Rhodospirillales, Firmicutes, Myxococcales and *Ralstonia*.

In endophytic communities, Proteobacteria (39.62–63.60%) was also the dominant phylum, followed by Actinobacteria (13.20–37.95%), Acidobacteria (2.64–10.46%), and Chloroflexi (1.88–10.80%) (Figure 4b). Dominant genera included *Acidothermus* (6.52–28.81%), *Acidibacter* (2.82–10.37%), *Ralstonia* (0.28–18.27%), *Bradyrhizobium* (2.44–9.55%), and *Thermosporothrix* (0.66–7.06%). Notably, *Ralstonia* and *Rhodococcus* were enriched in N_HN (13.79% and 3.46%), N_HB (18.27% and 7.09%), F_HN (15.37% and 1.29%), and F_YN (12.62% and 3.84%). In contrast, *Enterobacter* dominated the F_YN endophytic community, accounting for 19.82% of total sequences, whereas its abundance in other samples was below 1.44% (Figure 4d). In endophytic communities, 25 specialized OTUs were detected by SPEC–OCCU analysis, with F_FJ (7 OTUs) and F_GX (6 OTUs) harboring the highest numbers (Supplementary Figure S2b and Table S4). Out of the 25 specialized OTUs, 9 belonged to Proteobacteria (36%), 5 belonged to Actinobacteria (20%), and 4 belonged to Firmicutes (16%). LEfSe analysis identified 10 biomarker taxa in endophytic bacterial communities from nine different provenances (Figure 4f). These biomarkers mainly included Acidimicrobiales (F_FJ), Chloroflexi (F_AH), Ktedonobacteria (F_AH), Deltaproteobacteria (F_HN), Myxococcales (F_HN), Pseudomonadales (F_GX), Moraxellaceae (F_GX), and *Thermosporothrix* (F_SC). The principal bacterial biomarkers associated with native N_HB and N_HN were Burkholderiales, Nocardiaceae, *Rhodococcus,* Corynebacteriales, *Ralstonia*, *Burkholderia*, and *Streptomyces* (Figure 4h,j). In contrast, the predominant biomarkers associated with non-native F_HB and F_HN included Acidobacteria, Chloroflexi, *Frankia*, Bacteroidetes, Anaerolineaceae, Rhodospirillales, Polyangiaceae, and *Sorangium*.

### 3.5. Co-Occurrence Networks and Core Microbiota

**Figure 5:**
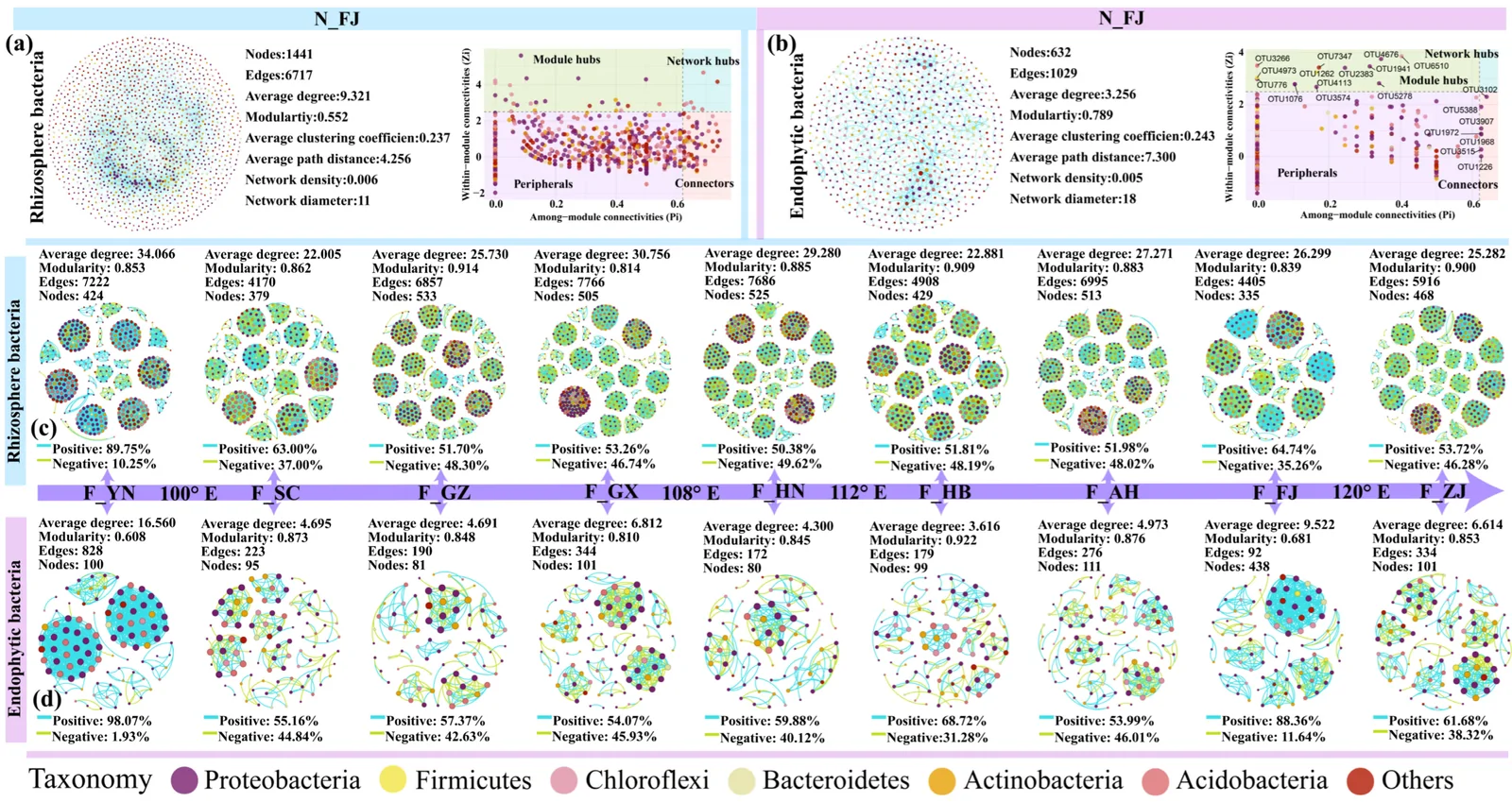
Co-occurrence networks and Zi–Pi analysis of root-associated bacterial communities of *T. wilfordii* across different provenances. (**a**,**b**) Global co-occurrence networks constructed from nine provenances cultivated in Fujian Province. (**c**,**d**) Subnetworks corresponding to the nine provenances. Each node represents an OTU, and node size is proportional to its degree. Nodes are colored according to phylum-level classification. Zi–Pi plots were used to identify keystone taxa. The thresholds for within-module connectivity (Zi) and among-module connectivity (Pi) were set at 2.5 and 0.62, respectively.

To evaluate differences in microbial interaction patterns among cultivation sites and provenances, 24 co-occurrence networks were constructed (Figure 5, Supplementary Figure S3 and Tables S5–S7). Network topology analysis showed that the global rhizosphere network contained more nodes, edges, and a higher average degree than the endophytic network (Figure 5a), indicating more frequent microbial interactions and greater overall complexity. The rhizosphere network also exhibited a shorter average path length and smaller network diameter, suggesting more efficient resource and information transfer. In contrast, the endophytic network displayed higher modularity and clustering coefficients (Figure 5b), reflecting stronger niche partitioning and greater compartmentalization of microbial interactions. Native populations from Hunan and Hubei exhibited distinct network characteristics in both rhizosphere and endophytic habitats. Topological differences between locations were more pronounced in rhizosphere networks than in endophytic networks (Supplementary Figure S3 and Table S7), indicating differential effects of provenance and habitat on microbial interaction patterns. Among provenance-specific subnetworks (Figure 5c,d), F_YN and F_GX exhibited the largest numbers of nodes, edges, and average degree values, indicating more complex network structures. Positive correlations predominated in F_YN and F_FJ networks, suggesting stronger cooperative interactions among taxa, whereas other networks displayed more balanced proportions of positive and negative correlations, indicative of a dynamic equilibrium between cooperation and competition. Network robustness analysis revealed that rhizosphere bacterial networks of F_AH, F_GZ, F_GX, and F_HN had relatively high stability (Supplementary Figure S4a). Network vulnerability increased significantly with increasing mean annual temperature (MAT) (*R*^2^ = 0.431, *p* = 0.033) (Supplementary Figure S4b), indicating reduced network stability under warmer climatic conditions. Endophytic networks showed generally lower robustness following random node removal, and no significant relationship with MAT was detected (Supplementary Figure S4d,e). Soil physicochemical properties and native climatic conditions were identified as the major drivers of network complexity. Models including both biotic and abiotic predictors explained 84.7% and 90.4% of the total variation in rhizosphere and endophytic network complexity, respectively (Supplementary Figure S4c,f). Core microbiota were identified based on within-module connectivity (Zi) and among-module connectivity (Pi). The rhizosphere and endophytic networks of the nine introduced provenances cultivated in the Fujian common garden contained 103 and 20 module hubs, respectively. In contrast, native Hunan and Hubei populations had fewer module hubs, with 6 and 0 in rhizosphere networks and 1 and 1 in endophytic networks, respectively (Supplementary Tables S5 and S6).

### 3.6. Environmental Drivers of Microbial Communities

**Figure 6:**
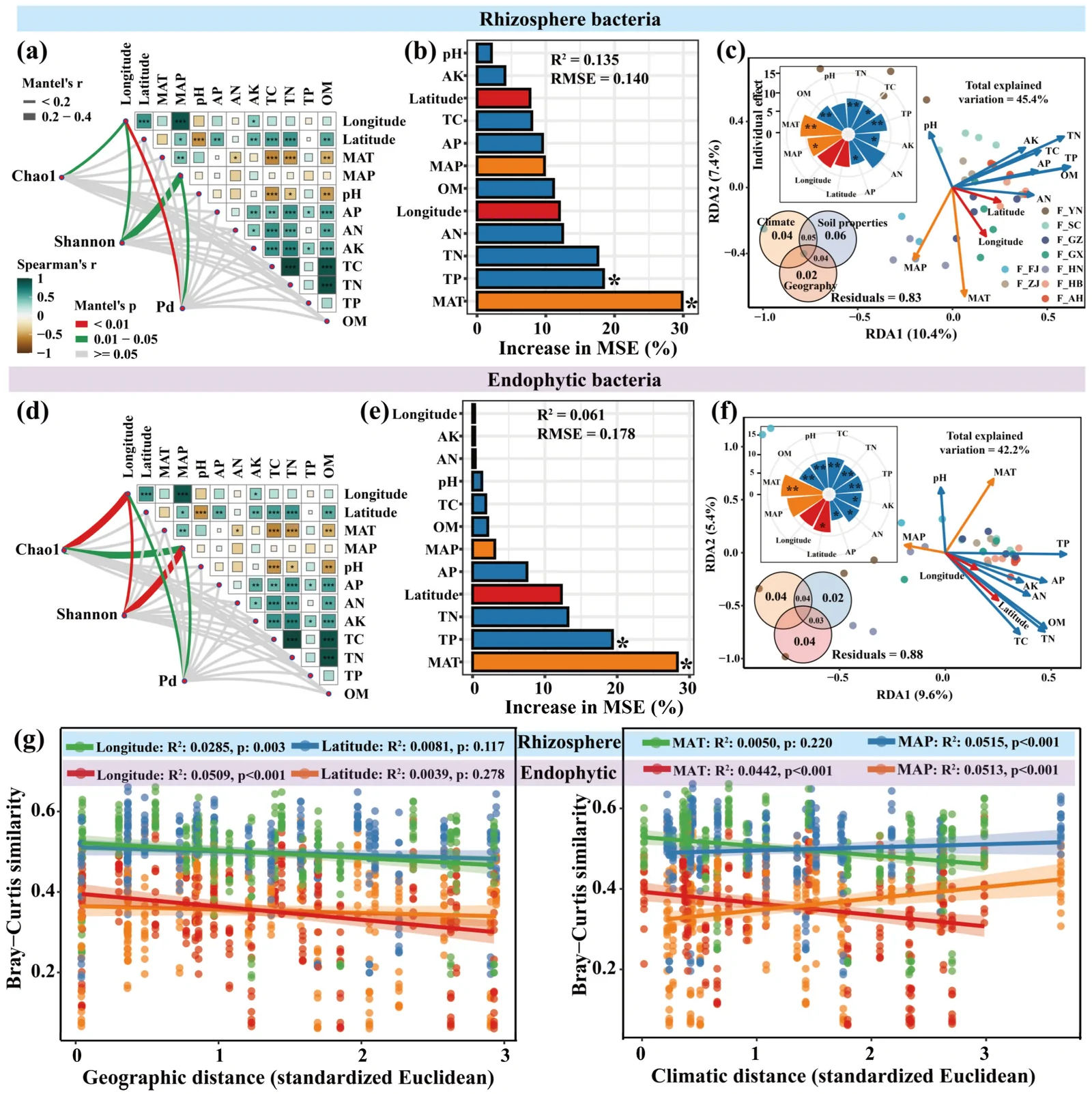
Effects of environmental factors on microbial communities. (**a**,**d**) Mantel tests evaluating the relationships between α-diversity indices and environmental variables. (**b**,**e**) Random forest analysis identifying key predictors of the first principal coordinate (PC1) from PCoA ordinations. Asterisk denotes significant differences (*p* < 0.05). (**c**,**f**) Redundancy analysis (RDA) illustrating the relationships between microbial community composition and environmental factors. Radar plots represent the independent explanatory power of each factor, including soil physicochemical properties at the planting site, climatic variables at native sites, and provenance geographic location. Venn diagrams depict variance partitioning analysis (VPA) quantifying the proportions of variation in microbial communities explained by native-site climatic variables, planting-site soil properties, and provenance geographic location. (**g**) Distance–decay relationships showing Bray–Curtis similarity as a function of geographic and climatic distances among provenances. Solid lines represent ordinary least squares (OLS) regression fits. (* *p* < 0.05, ** *p* < 0.01, and *** *p* < 0.001).

The α-diversity indices of both rhizosphere and endophytic bacterial communities were significantly correlated with the longitude and mean annual precipitation (MAP) of host native origins (*p* < 0.05; Figure 6a,d). Random forest analysis identified MAT and TP as the primary predictors of community variation along the PC1 in both rhizosphere and endophytic bacterial communities (Figure 6b,e). DDRs analysis was used to assess whether root-associated bacterial communities retained signatures of environmental conditions from their native habitats. Significant negative relationships were observed between Bray–Curtis similarity and geographic distance among host native origins. Specifically, community similarity decreased significantly with increasing longitudinal distance among host native origins for both rhizosphere (*R*^2^ = 0.0285, *p* = 0.003) and endophytic bacterial communities (*R*^2^ = 0.0509, *p* < 0.001), in addition, rhizosphere community similarity declined significantly with increasing MAP distance *R*^2^ = 0.0515, *p* < 0.001) (Figure 6g). For endophytic communities, Bray–Curtis similarity decreased significantly with increasing climatic distance based on both MAT (*R*^2^ = 0.0442, *p* < 0.001) and MAP (*R*^2^ = 0.0513, *p* < 0.001) (Figure 6g). Among the environmental variables examined, MAP explained the largest proportion of variation, followed by longitude and MAT. RDA further demonstrated significant associations between environmental factors and root-associated bacterial community structure (Figure 6c,f). The first two RDA axes explained 17.8% and 15.0% of the total variation in rhizosphere and endophytic bacterial communities, respectively. For rhizosphere communities, MAT, TN, and TP were the primary environmental drivers (Figure 6c). In contrast, endophytic community variation was mainly associated with MAT, OM, pH, TC, TN, and TP (Figure 6f). VPA showed that soil properties, native climatic conditions, and native geographic location explained 6%, 4%, and 2% of the variation in rhizosphere bacterial community structure, respectively (Figure 6c). For endophytic bacterial communities, the corresponding contributions were 2%, 4%, and 4%, respectively (Figure 6f).

**Figure 7:**
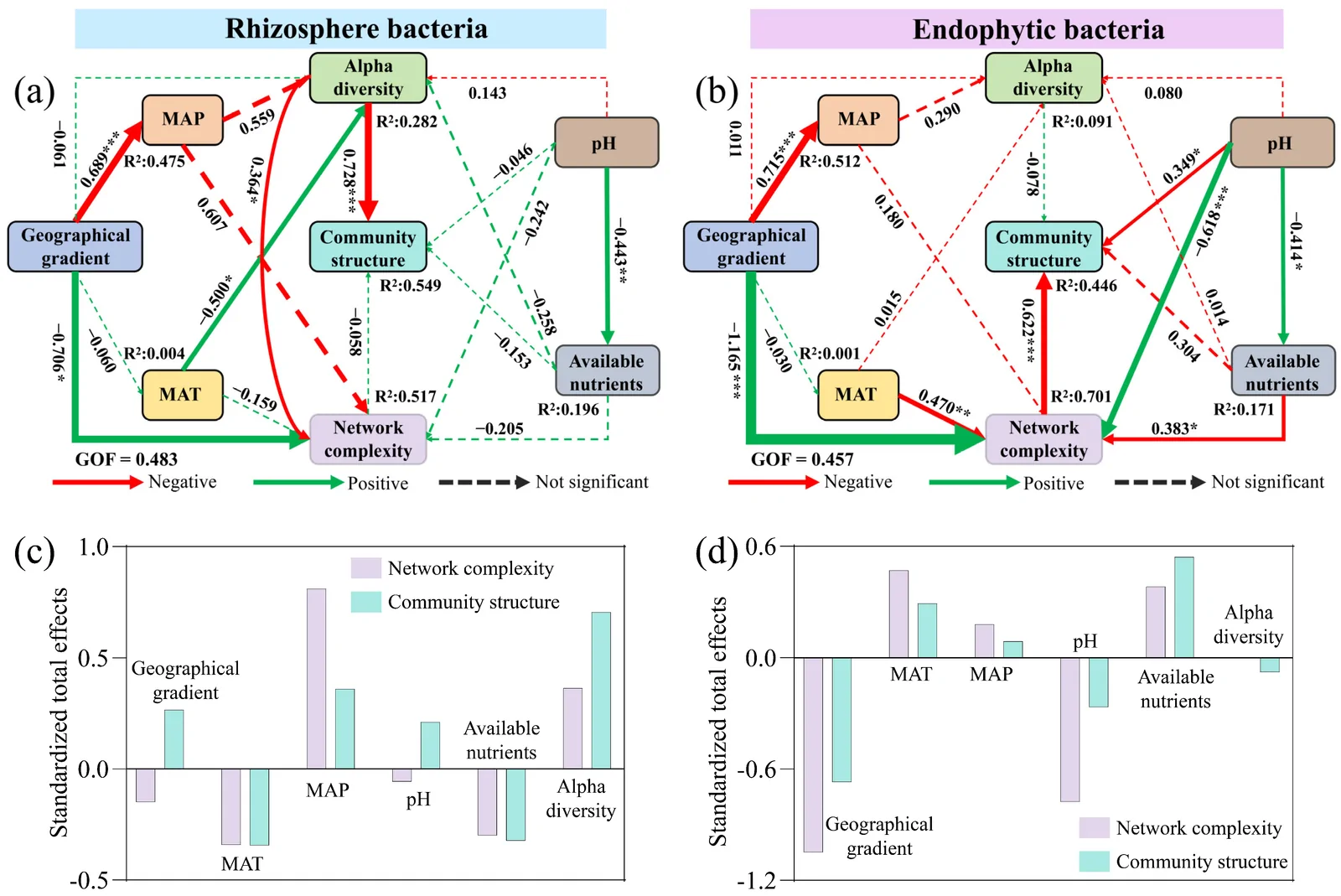
Structural equation modeling (SEM) of the effects of environmental factors on bacterial community structure. (**a**,**b**) SEM path diagram. Standardized path coefficients are shown on arrows. *R*^2^ represents the proportion of variance explained by the model. Significance levels are indicated as * *p* < 0.05, ** *p* < 0.01, and *** *p* < 0.001. (**c**,**d**) Standardized total effects of each individual driver on bacterial communities derived from the SEM.

Structural equation modeling (SEM) revealed the direct and indirect effects of geographic location, historical climate, and soil properties on bacterial α-diversity, community structure, and network complexity. For rhizosphere bacterial communities (Figure 7a), MAT exerted a significant effect on α-diversity. Both α-diversity and geographic location significantly influenced community structure, whereas α-diversity also had a significant positive effect on network complexity. Total effect analysis indicated that MAP and α-diversity exerted the strongest overall influences on network complexity and community structure, respectively (Figure 7c). For endophytic bacterial communities (Figure 7b), network complexity was significantly affected by geographic location, MAT, soil pH, and soil nutrient availability, whereas community structure was significantly influenced by soil pH and network complexity. Total effect analysis showed that geographic location had the strongest overall effect on both network complexity and community structure (Figure 7d).

### 3.7. Functional Characteristics of Bacterial Communities

**Figure 8:**
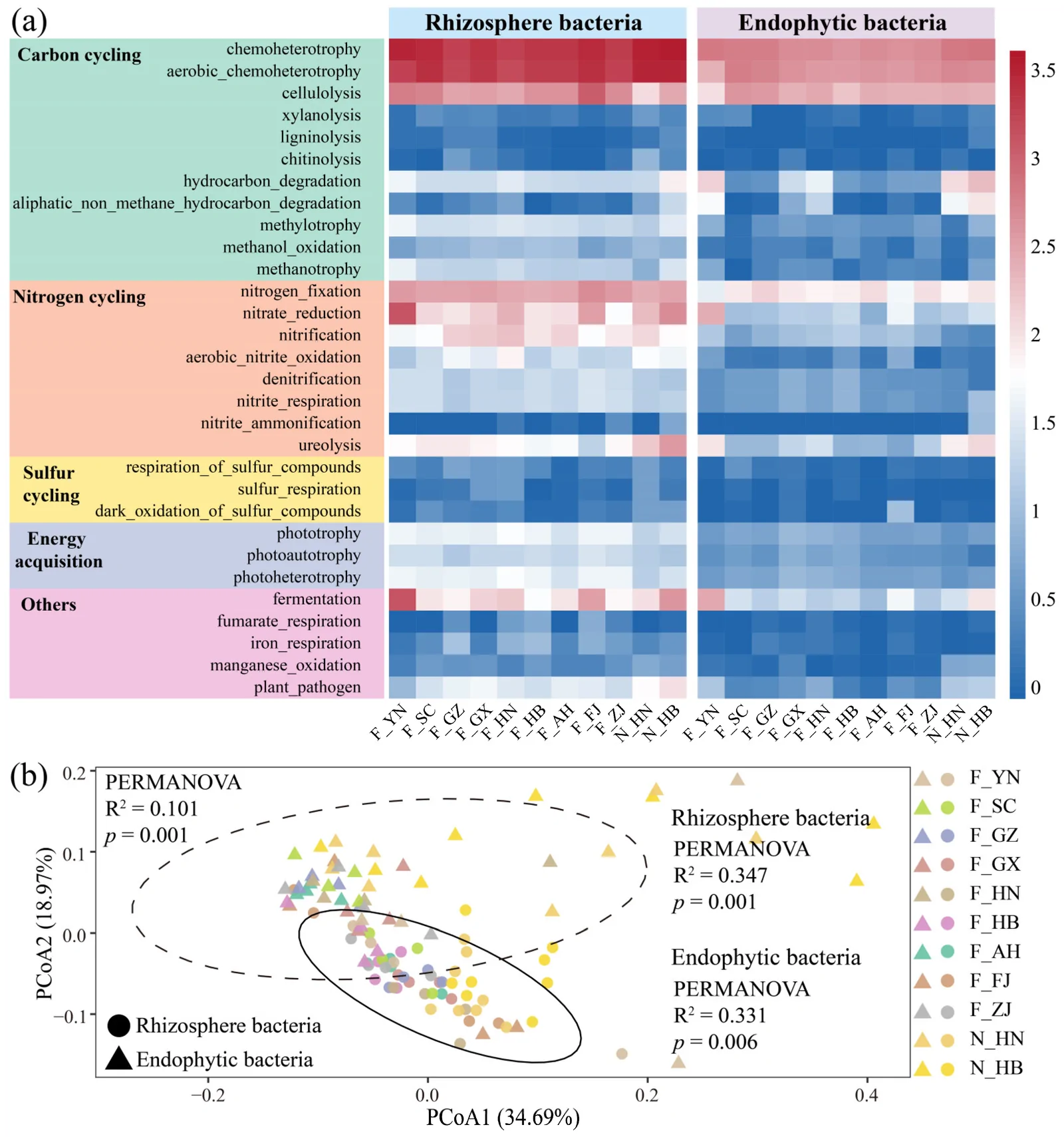
Functional prediction of bacterial communities based on FAPROTAX. (**a**) Heatmap showing the relative abundances of the 30 predicted functional groups of bacterial communities inferred using the FAPROTAX database. (**b**) Principal coordinates analysis (PCoA) based on Bray–Curtis dissimilarity illustrating differences in functional composition between rhizosphere and endophytic bacterial communities. Colors indicate different provenances, and shapes represent ecological compartments.

Functional annotation based on the FAPROTAX database revealed substantial functional diversity in both rhizosphere and endophytic bacterial communities associated with *T. wilfordii* (Figure 8). Overall, chemoheterotrophy, aerobic chemoheterotrophy, cellulolysis, and nitrogen fixation were the dominant functional groups in both ecological niches (Figure 8a). In rhizosphere bacterial communities, the relative abundances of multiple functional groups differed significantly among provenances. Within carbon-cycling functions, hydrocarbon degradation, aliphatic non-methane hydrocarbon degradation, chemoheterotrophy, aerobic chemoheterotrophy, and cellulolysis exhibited significant variation. Among nitrogen-cycling functions, nitrification, aerobic nitrite oxidation, and ureolysis differed significantly among provenances. Within sulfur-cycling functions, only dark oxidation of sulfur compounds showed significant variation. In addition, potential plant pathogen varied significantly among provenances. Aerobic chemoheterotrophy, chemoheterotrophy, plant pathogen, dark oxidation of sulfur compounds, ureolysis, and aliphatic non-methane hydrocarbon degradation were significantly enriched in the native populations N_HN and N_HB (*p* < 0.05). Aerobic nitrite oxidation was significantly enriched in F_HN, whereas cellulolysis showed relatively high abundance in F_FJ. Nitrification was significantly more abundant in F_GZ, F_GX, and F_HN than in other provenances (*p* < 0.05; Supplementary Figure S5). In contrast, fewer functional groups differed significantly among provenances in endophytic bacterial communities. Only aliphatic non-methane hydrocarbon degradation, hydrocarbon degradation, nitrification, and ureolysis exhibited significant variation among provenances (Supplementary Figure S5). Overall, these findings indicate that host provenance significantly influences the functional composition of root-associated bacterial communities in *T. wilfordii*, with stronger effects observed in the rhizosphere than in the endosphere.

PCoA based on functional profiles further revealed clear differentiation in bacterial functional structure (Figure 8b). Functional composition differed significantly between rhizosphere and endophytic niches (PERMANOVA, *R*^2^ = 0.101, *p* = 0.001). Significant separation among provenances was also observed in rhizosphere (*R*^2^ = 0.347, *p* = 0.001) and endophytic bacterial communities *R*^2^ = 0.331, *p* = 0.006). Collectively, these results demonstrate that both ecological niche and host provenance significantly shape the functional structure of bacterial communities associated with *T. wilfordii*, with provenance representing a major driver of functional differentiation.

## 4. Discussion

### 4.1. Host Genotype-Driven Changes in Microbial Diversity and Community Structure

Plant genotypes can influence various factors, including root architecture and the composition of root exudates, which may reshape the rhizosphere niche and thereby alter root-associated microbiomes [49]. In the rhizosphere, we observed significant differences in bacterial richness, diversity, and community structure among different provenances under identical environmental conditions (Figure 3a–c,g), indicating that genetic variation among provenances plays a critical role in shaping rhizosphere microbiomes. These findings are consistent with previous studies demonstrating that host genotype can alter the composition and structure of rhizosphere microbial communities [19,21,50]. Ricks et al. [51] reported significant differences in microbial composition among *Brachypodium distachyon* genotypes originating from different locations, in which host genetic background explained approximately 20–30% of the observed variation. Similarly, in the present study, 27.0% of the variation in rhizosphere bacterial community composition could be explained by host genetic background (Figure 3g). Network analysis further revealed distinct co-occurrence network structures of rhizosphere bacteria among different provenances (Figure 5), highlighting the influence of provenance on bacterial interactions within the rhizosphere. Root exudates function not only as sources of carbon compounds and energy but also as signaling molecules that regulate the assembly of rhizosphere microbial communities [52]. Plants from different geographic provenances have undergone long-term adaptation to local environmental conditions, resulting in the evolution of distinct regulatory mechanisms for secondary metabolism. Previous studies on *T. wilfordii* demonstrated significant differences in the accumulation of medicinal secondary metabolites among clonal plants from different provenances [53]. These differences may reflect variation in the composition or quantity of root exudates, thereby affecting microbial colonization, competition, and activity in the rhizosphere.

Compared with rhizosphere bacterial communities, the diversity of endophytic bacterial communities among different provenances exhibited relatively conserved diversity patterns; however, their community structures still showed significant differences, and these differences were even more pronounced (Figure 3d–f,h). Meanwhile, the co-occurrence network structures of root endophytic bacterial communities also varied among different provenances (Figure 5). Ji et al. [54] also observed a greater effect of plant genetic variation on the root endophytic microbiome than rhizosphere compartments. Brown et al. [55] further noted pronounced host filtering, in which host genetics drove the endophytic microbial composition, whereas the rhizosphere communities were primarily shaped by soil origin. These findings suggest that the host plant genetic background plays a more important role in the assembly of the endosphere microbiome than that of the rhizosphere compartment. According to a two-step model for microbiome colonization [56], root exudates initially drive a shift in the microbial community composition within rhizosphere, and subsequently, plant genetic factors regulate microbial entry into internal root tissues. To enter the internal structures of plants, microorganisms must overcome the host immune barriers [57]. Genetic variation associated with plant immune responses is widespread [58]. Therefore, genotype-driven alterations in plant internal tissue immune mechanisms can significantly enhance host genetic selection pressure on microbial colonization [59]. In addition, plant genotypes can selectively regulate endophytic microorganisms through specific metabolic pathways. For example, Lignin engineering in field-grown poplar trees reshaped the structure of root endophytic bacterial communities rather than rhizosphere microbiomes by altering the accumulation of phenolic compounds in roots [60].

Our study further revealed that, for both rhizosphere and root endophytic bacterial communities, plants growing in their native habitats (N_HB and N_HN) exhibited significantly lower community richness and diversity compared with plants of the same provenances transplanted to novel non-native locations (F_HB and F_HN) (Figure 3a–f). Native plants have co-evolved over long periods with specific soil microbial communities in their original habitats, potentially establishing stable and specialized symbiotic relationships characterized by lower microbial diversity but higher functional efficiency. In contrast, non-native plants introduced into new environments may lack local adaptation, creating ecological niches that are initially more accessible for microbial colonization, thereby facilitating the recruitment of more diverse bacterial communities [61]. The high microbial diversity is involved in plant growth promotion, plant defense, and soil-borne disease suppression [62]. The increased diversity of root-associated microbial communities may facilitate the adaptation of non-native plants to novel environments. However, it should be recognized that increased microbial community diversity is not a universal phenomenon, as this process is jointly determined by multiple factors, including host genetic background, environmental conditions of the introduced soil, and ecological strategies of microbial communities themselves. In particular, the soil microbial pool and physicochemical properties of the transplant site play dominant roles in shaping root-associated microbiomes.

Despite the significant provenance effect, neutral community model analysis revealed that stochastic processes played a dominant role in bacterial community assembly (Supplementary Figure S1d–g). Random dispersal, ecological drift, and demographic stochasticity largely determine microbial distribution patterns. Such results suggest that community assembly in *T. wilfordii* root-associated microbiomes is not solely driven by environmental filtering or host selection but is substantially influenced by neutral ecological processes. In various root-associated microhabitats, including the rhizosphere of halophytic plants [63], inter-root soils of cultivated *Acanthopanax senticosus* across different geographic locations [64], and rhizosphere soils of *Phoebe bournei* plantations under different fertilization regimes [65], microbial community structures were significantly influenced by neutral processes, such as ecological drift and dispersal limitation. Notably, the endophytic environment generally exhibits stronger host control. For example, soybean root-associated endophytic microbial communities are driven by homogeneous selection regulated by plant developmental stages [66]. In the present study, the migration rate of endophytic bacteria was lower than that of rhizosphere bacteria. This pattern reflects the physical and biological barriers imposed by plant tissues, which restrict microbial colonization and movement. Consequently, only a subset of the rhizosphere microbiota can successfully establish within plant tissues, leading to stronger dispersal limitation and enhanced host-mediated filtering in endophytic communities [67].

### 4.2. Compositional Characteristics of Microbial Communities

Both rhizosphere and endophytic bacterial communities of *T. wilfordii* were consistently dominated by Proteobacteria, followed by Actinobacteria, Acidobacteria, and Chloroflex (Figure 4a,b), indicating a conserved phylogenetic composition across provenances. These four phyla have been widely identified as the dominant bacterial community in the root environments of diverse plants, such as maize, *Acacia*, oak, bamboo, oat, cactus, rice, and potato [34,68,69,70]. Their relative abundances within bacterial communities depend on specific environmental conditions [71]. Proteobacteria, characterized by rapid growth rates and efficient utilization of root-derived carbon compounds [72], are among the dominant colonizers in root-associated habitats. Many members of this phylum exhibit plant growth-promoting capabilities, including biological nitrogen fixation, phytohormone production, and pathogen suppression [73,74]. Actinobacteria are considered key microorganisms involved in the degradation of complex organic substrates, including lignin, cellulose, and chitin [75,76], and play essential roles in nutrient mineralization within forest ecosystems. Many Actinobacteria taxa can also produce antimicrobial compounds that inhibit plant pathogens [77]. Acidobacteria and Chloroflexi are generally associated with nutrient-poor forest soils and participate in nutrient cycling processes [78,79]. Therefore, the dominant bacterial taxa consistently detected among different provenances may play critical ecological roles in the growth, nutrient acquisition, and environmental adaptation of *T. wilfordii*. LEfSe analysis identified provenance-specific bacterial biomarkers in both rhizosphere and endophytic compartments, including Frankiales, *Acidothermus*, Burkholderiales, *Streptomyces*, Acidimicrobiales, Ktedonobacteria, Pseudomonadales, and *Thermosporothrix* (Figure 4e–f). These taxa generally possess functions related to organic matter decomposition, nitrogen fixation, nitrification, and pathogen suppression, and may play important roles in maintaining nutrient cycling processes, including carbon, nitrogen, and sulfur cycling, within soil–plant systems, particularly under acidic or nutrient-limited conditions. Collectively, these findings suggest that plants from different provenances may enhance nutrient acquisition and environmental adaptation by selectively recruiting specific root-associated functional microbial groups.

Although plant growth-promoting (PGP) functions are distributed among diverse microbial groups [80], we still observed that plants growing in their native habitats (N_HB and N_HN) recruited more plant growth-promoting bacteria in the root-associated regions than transplanted non-native plants (F_HB and F_HN), including *Pseudomonas*, *Arthrobacter*, *Burkholderia*, *Streptomyces*, *Massilia*, Nocardiaceae, and *Novosphingobium* (Figure 4g–j). These microorganisms have been widely demonstrated to possess plant growth-promoting traits, such as phosphate solubilization, nitrogen fixation, phytohormone production, siderophore synthesis, pathogen suppression, and enhancement of plant stress tolerance [13,81,82,83,84,85,86,87,88]. In a previous study, more plant growth-promoting bacteria with nitrogen fixation, phosphate solubilization, and IAA-producing abilities, including *Pseudomonas*, *Burkholderia*, and *Enterobacter*, were isolated from the rhizosphere and endosphere of native *T. wilfordii* plants [34]. These findings support the enrichment of more abundant PGP bacteria in root-associated microbiomes of *T. wilfordii* growing in native habitats. Rodrigo et al. [89] demonstrated that although soil type or geographic location exerts a stronger influence on rhizosphere bacterial community structure than plant species, the plant growth-promoting functions of rhizosphere bacteria are primarily selected by plant species. The rhizosphere of *Ceanothus velutinus* treated with native soil harbored more PGP bacteria than those treated with greenhouse soil [90]. Therefore, enhanced recruitment of PGP bacteria may be attributed to the long-term adaptation of native plants to their local environments. In contrast, modern plant breeding has reduced the capacity of crops to select and host PGP bacteria [91,92]. The superior environmental adaptability of native plants may also originate from their stronger capacity to selectively recruit microorganisms with PGP functions. Traits previously considered to be completely determined by plants themselves, such as stress tolerance, disease resistance, and nutrient acquisition, are increasingly recognized as emergent phenotypes resulting from adaptive microbiome recruitment and selection of microbiomes by plants [93]. Overall, as demonstrated by previous studies, the rhizosphere microbiome of native plants represents a valuable reservoir of PGP bacteria, which can be employed as biofertilizers and biostimulants [90].

### 4.3. Historic Climate Drivers of Microbial Community Differentiation

The root-associated microbiome is shaped by multiple factors root exudates, soil pH, nutrient characteristics, and other environment variables, such as geographical location, temperature and water availability [56,94,95,96,97,98]. This study demonstrated that the longitude of the native habitats and mean annual precipitation were the primary environmental factors shaping the diversity of root-associated microbial communities in *T. wilfordii* from different provenances (Figure 6a,d). The Bray–Curtis similarity significantly decreased with increasing geographical distance in longitude and divergence in precipitation regimes (Figure 6g). Although longitude itself is not a direct ecological driver, it serves as a robust proxy for the pronounced east–west precipitation gradient across southern China, where MAP decreases progressively from coastal to inland regions. Therefore, the significant effects of longitude and mean annual precipitation may be attributed to the east–west distribution pattern of native habitats of the sampled provenances. Mean annual precipitation has also been identified as the most important environmental constraint regulating microbial community assembly in the soil–root continuum across the Chinese Loess Plateau [98]. In this study, bacterial community diversity in both soils and roots of desert steppe ecosystems was lower than that observed in temperate and warm-temperate grasslands. These findings support the view that water availability and precipitation play critical roles in determining bacterial distribution patterns [99]. Furthermore, our results indicate that root-associated bacterial communities of *T. wilfordii* clones from different provenances retained signatures of historical climate from their native habitats even after cultivation under common environmental conditions. This observation is consistent with the findings of Ricks et al. [51]. Their study revealed that 40 *Brachypodium distachyon* genotypes originating from distinct geographic locations maintained microbial signatures associated with their native environments despite being grown under identical conditions. A proportion of the variation in microbial community composition was strongly associated with historical nitrogen availability at the plant origin sites, and genotypes from nitrogen-poor habitats exhibited significant enrichment of nitrogen-fixing bacterial communities. These observations suggest that multiple heritable plant traits involved in microbiome recruitment may be shaped by selection imposed by abiotic environmental conditions. Consequently, when plants are cultivated outside their native habitats, their associated microbiomes may still preserve ecological signatures reflecting the historical environments in which the plants evolved [51].

### 4.4. Functional Differentiation of Root-Associated Microbiomes

The quality of medicinal plants is shaped by interactions between host genotype and environmental factors, including soil properties and climatic conditions, which indirectly influence bioactive compound accumulation through microbiome-mediated processes. Consistent with previous studies in medicinal plants such as *Salvia miltiorrhiza*, *Curcuma* spp., *Andrographis paniculata*, and *Dendrobium officinale* [100,101,102,103,104], our results demonstrate significant provenance-dependent differentiation in microbial community structure and function (Figure 8). Functional prediction based on FAPROTAX revealed that chemoheterotrophy, aerobic chemoheterotrophy, cellulolysis, and nitrogen fixation dominated both rhizosphere and endophytic communities. These functions represent key processes in carbon and nitrogen cycling, consistent with typical plant–soil microbial interaction systems [105]. Geographic location and soil conditions predominantly shape the phylogenetic composition of rhizosphere bacterial communities, while plant genotype plays a decisive role in governing the functional attributes of the associated rhizosphere microbiome [89]. Plant genotype likely modulates microbial recruitment through differences in root exudate chemistry, thereby shaping assembly of functional microbial guilds [3]. We observed significant differences in the relative abundances of some potential functional groups associated with carbon, nitrogen, and sulfur cycling among provenances within the rhizosphere and root endophytic compartments. Significant provenance-dependent variation in nitrogen cycling-related functions, e.g., nitrification and ureolysis, suggests that host genetic background may reshape microbial nutrient transformation potential and thereby indirectly influence plant–microbe resource exchange and secondary metabolism [106]. Different from community structure, functional differentiation was more evident in rhizosphere communities than in endophytic communities in the present study. This pattern may be attributed to the direct exposure of rhizosphere to soil nutrient availability, physicochemical conditions, and environmental microbial reservoirs. Consequently, rhizosphere communities may exhibit greater functional plasticity in response to differences among plant provenances. In contrast, endophytic communities experience stronger selective filtering imposed by host tissues. Such selective constraints may reduce broad-scale functional variation. However, this difference does not imply a reduced functional importance of endophytic microorganisms, which play essential roles in nutrient exchange, stress adaptation, and regulation of host physiological processes through intimate interactions with plant tissues. In addition, enrichment of plant pathogen-associated functional groups in some provenances suggests that functional differentiation may also be linked to plant health status and stress responses [107]. Overall, provenance-driven functional restructuring of microbial communities may represent a key ecological mechanism linking environmental adaptation and medicinal compound formation.

Although this study provides new insights into the role of provenance and environmental history in shaping root-associated bacterial communities of *T. wilfordii*, several limitations should be acknowledged. First, 16S rRNA gene amplicon sequencing provides limited taxonomic resolution and cannot directly reveal microbial functional activities. Future studies integrating metagenomic and metabolomic approaches are needed to better understand the functional consequences of microbiome differentiation. Second, although the common-garden experiment minimized environmental heterogeneity, host-related factors such as root exudates and genetic variation were not investigated, which may contribute to provenance-specific microbial recruitment. Third, the observed patterns mainly reflect ecological associations, and further manipulative experiments are required to establish causal links between plant provenance, environmental legacy, and microbiome assembly.

## 5. Conclusions

This study investigated whether host provenance influences the assembly and functional differentiation of root-associated bacterial communities in *T. wilfordii* under common-garden conditions, and evaluated the relative contributions of host genetic background, environmental legacy, and ecological processes in shaping microbiome variation. Both rhizosphere and root endophytic bacterial communities exhibited significant differentiation among *T. wilfordii* provenances, indicating that host genetic variation represents an important factor influencing microbial recruitment. Although rhizosphere communities were more strongly affected by external environmental conditions and microbial dispersal, endophytic bacterial communities exhibited stronger provenance-related differentiation, suggesting enhanced host filtering within internal plant tissues. Stochastic processes, particularly ecological drift and dispersal-related processes, dominated bacterial community assembly, while deterministic selection associated with host genotype played a more prominent role in shaping endophytic communities. In comparison with those transplanted outside native habitats, native plants were identified harboring more plant growth-promoting bacteria within their roots. Microbial interaction networks varied substantially among provenances, with network complexity and stability being jointly regulated by host origin, soil properties, and climatic factors. Functional prediction analysis revealed significant provenance-dependent differences in bacterial metabolic potential, particularly in carbon and nitrogen cycling processes. Geographic and climatic variables of plant native origins including longitude, precipitation and temperature were closely associated with microbial diversity, community structure and network stability, indicating potential environmental legacy effects. Overall, our findings demonstrate that the root-associated microbiome of *T. wilfordii* is shaped by the combined influences of host genetics background, environmental history, and ecological filtering. These results provide a theoretical basis for further investigations into plant–microbe interactions, provenance selection, and microbial resource exploitation in plant breeding and cultivation.

## Data Availability

The original contributions presented in this study are included in the article/Supplementary Materials. Further inquiries can be directed to the corresponding authors.

## Supplementary Materials

The following supporting information can be downloaded at: https://www.mdpi.com/article/10.3390/microorganisms14081694/s1, Figure S1: PCoA and community assembly processes of root-associated bacterial communities; Figure S2: SPEC–OCCU analysis based on the top 800 most abundant OTUs; Figure S3: Co-occurrence networks and Zi–Pi plots of bacterial communities in N_HN and N_HB native sites; Figure S4: Network stability and environmental drivers; Figure S5: Differences in the relative abundance of predicted functional groups among provenances; Table S1: Sample information of *Tripterygium wilfordii*; Table S2: Soil physicochemical properties of the sampling sites; Table S3: Specialist taxa identified in rhizosphere bacterial communities using the SPEC-OCCU framework; Table S4: Specialist taxa identified in endophytic bacteria communities using the SPEC-OCCU framework; Table S5: Keystone taxa identified from co-occurrence network analysis of rhizosphere bacterial communities in the three planting sites; Table S6: Keystone taxa identified from co-occurrence network analysis of endophytic bacteria communities in the three planting sites; Table S7: Topological properties of co-occurrence networks in N_HN and N_HB native sites.
