# Supplementary material for: Linking Host Provenance, Microbial Assembly, and Environmental Legacy Effects in the Root-Associated Microbiome of Tripterygium wilfordii Hook f
Source: Microorganisms. 2026 Aug 1;14(8):1694. doi: 10.3390/microorganisms14081694 (PMC13515602; doi:10.3390/microorganisms14081694)
Supplement: Supplementary file 1 [file microorganisms-14-01694-s001.zip › Supplementary_Figures.pdf]

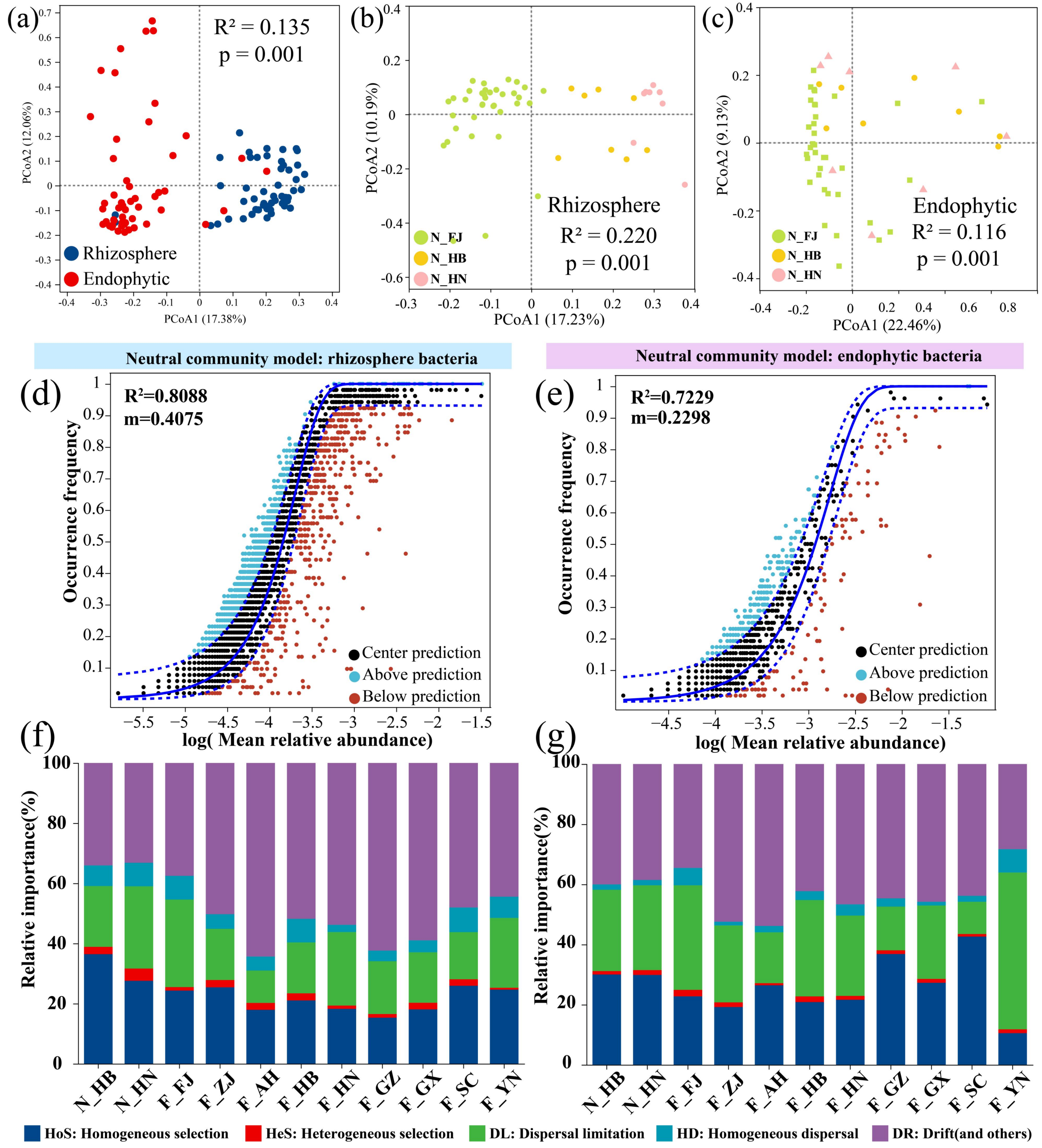

Supplementary Figure S1. PCoA and community assembly processes of root-associated bacterial communities. Principal coordinate analysis (PCoA) of bacterial communities based on Bray–Curtis dissimilarity: (**a**) rhizosphere and endophytic communities; (**b, c**) communities across the three planting sites. (**d, e**) Neutral community model (NCM) analysis of rhizosphere and endophytic bacterial communities. The solid bule line represents the best fit to the neutral model, and dashed lines indicate 95% confidence intervals. R^2 represents the goodness of fit of the NCM. (**c, d**) Relative contributions of ecological processes to bacterial community assembly based on the phylogenetic bin-based null model analysis (iCAMP).

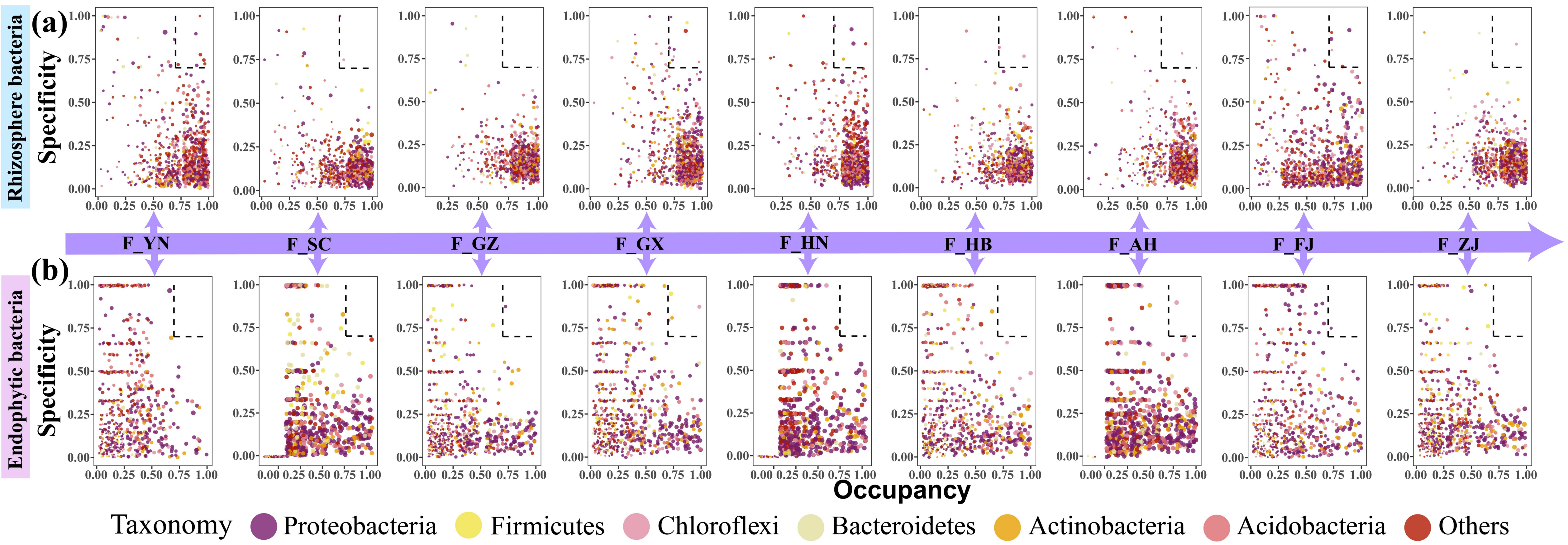

Supplementary Figure S2. SPEC–OCCU analysis based on the top 800 most abundant OTUs. (**a**) Rhizosphere bacterial communities. (**b**) Endophytic bacterial communities. Specialist taxa were defined as OTUs with specificity and occupancy ≥ 0.7. The x-axis represents occupancy, and the y-axis represents specificity.

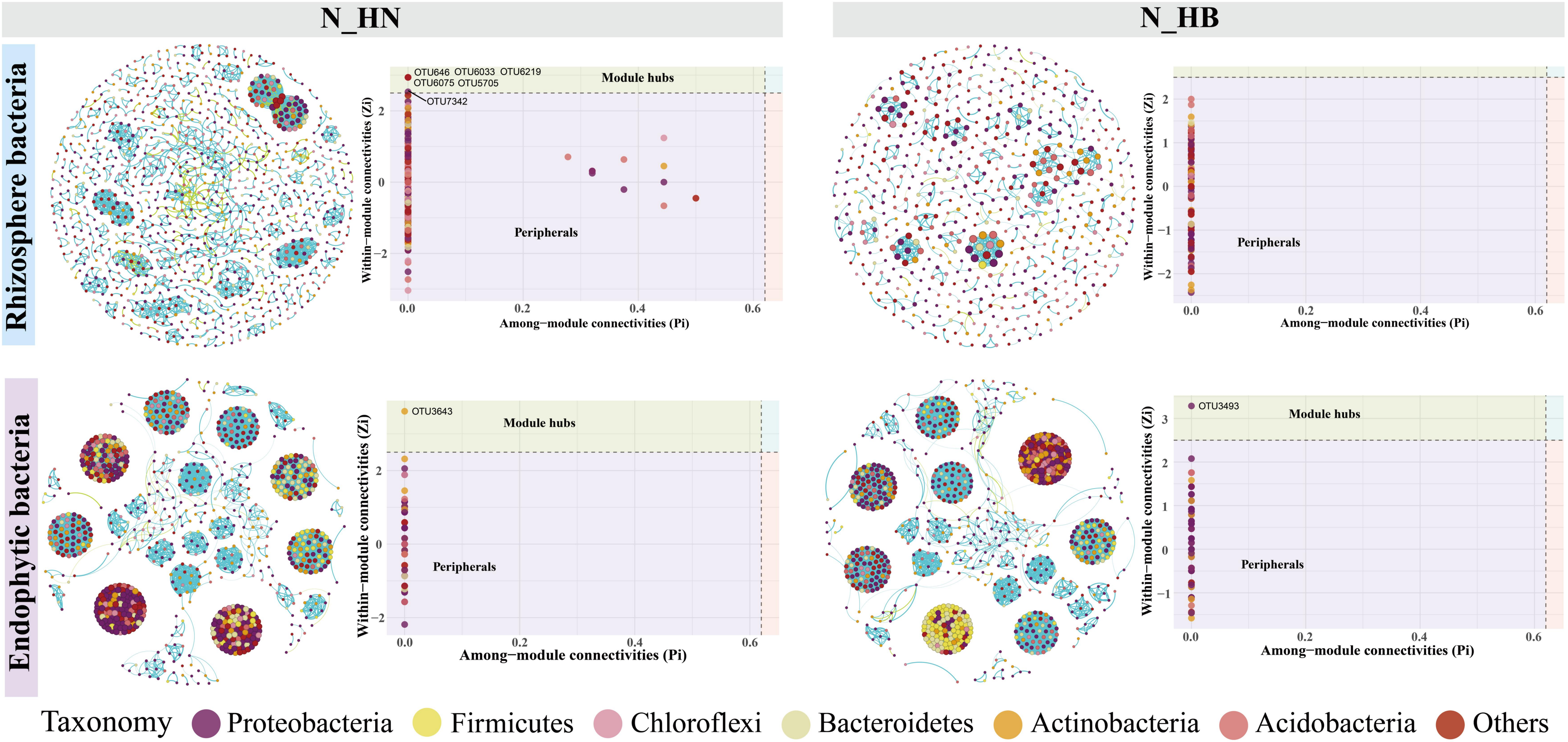

Supplementary Figure S3. Co-occurrence networks and Zi–Pi plots of bacterial communities in N_HN and N_HB native sites. Each node represents an OTU, with node size proportional to degree and color indicating phylum affiliation. Keystone taxa were identified using Zi = 2.5 and Pi = 0.62 thresholds.

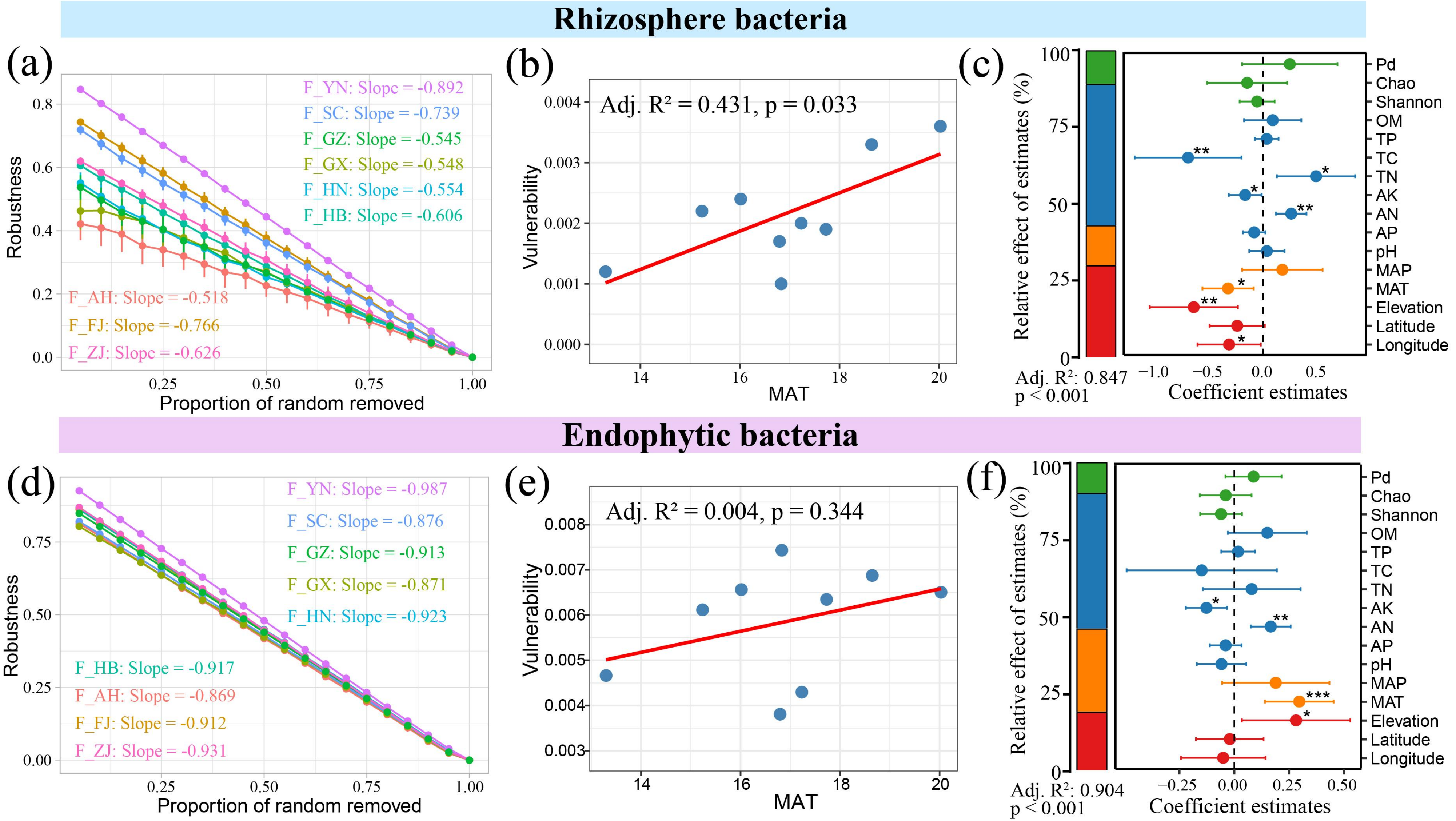

Supplementary Figure S4. Network stability and environmental drivers: (**a, d**) Robustness analyses of bacterial co-occurrence networks across the nine provenances; (**b, e**) Linear relationships between network properties and MAT; (**c, f**) Relative importance of environmental variables explaining network complexity.

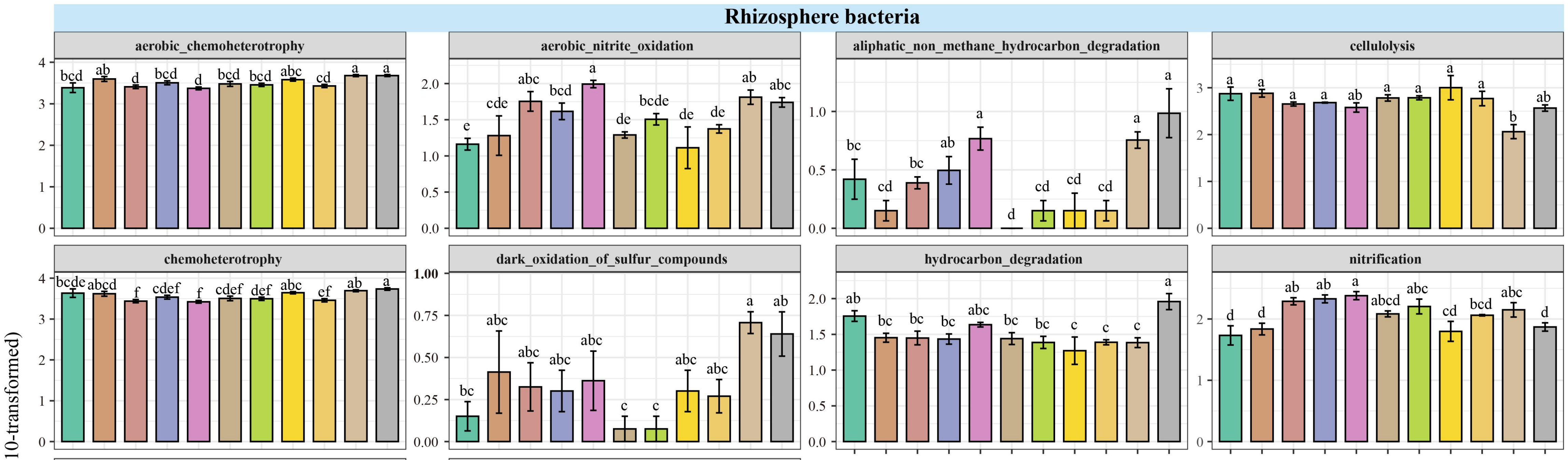

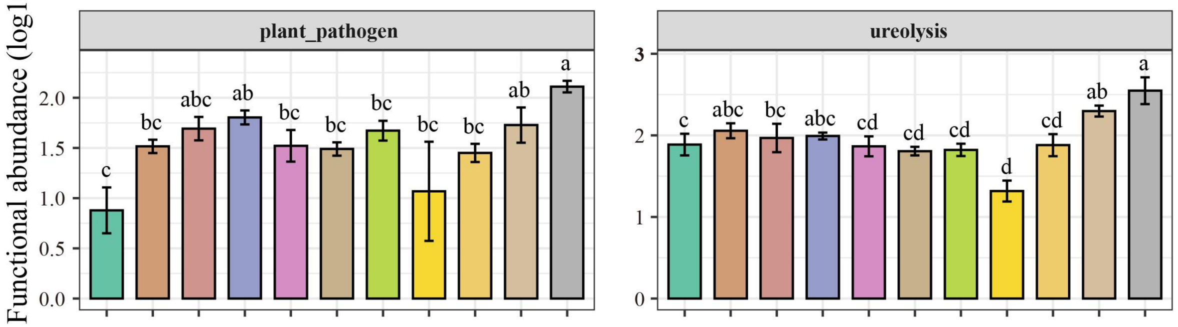

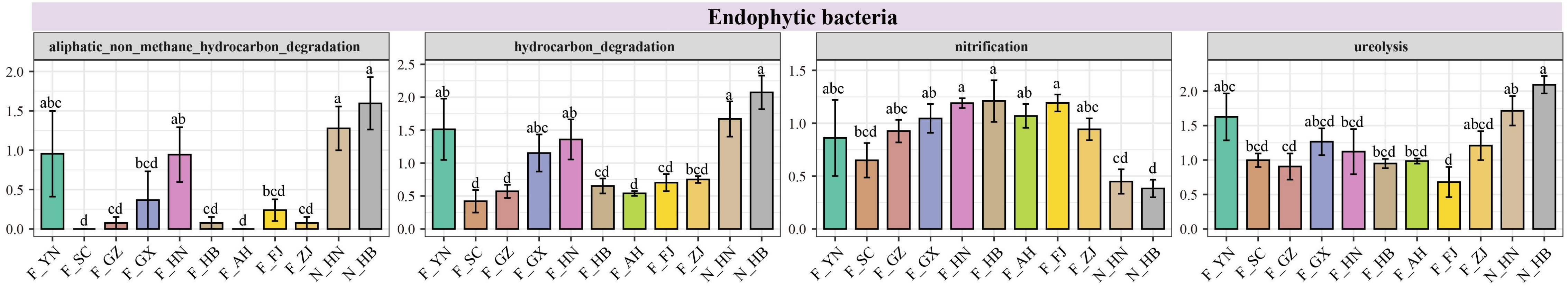

Supplementary Figure S5. Differences in the relative abundance of predicted functional groups among provenances. Significant differences were determined using the Kruskal–Wallis test with Benjamini–Hochberg (BH) correction ($p < 0.05$). Different letters indicate significant differences among provenances.
